# Epigenetic reprogramming enhances the therapeutic efficacy of osteoblast‐derived extracellular vesicles to promote human bone marrow stem cell osteogenic differentiation

**DOI:** 10.1002/jev2.12118

**Published:** 2021-07-07

**Authors:** Kenny Man, Mathieu Y. Brunet, Maria Fernandez‐Rhodes, Soraya Williams, Liam M. Heaney, Lee A. Gethings, Angelica Federici, Owen G. Davies, David Hoey, Sophie C. Cox

**Affiliations:** ^1^ School of Chemical Engineering University of Birmingham Birmingham UK; ^2^ School of Sport, Exercise and Health Sciences Loughborough University Loughborough UK; ^3^ Waters Corporation Stamford Avenue Wilmslow UK; ^4^ Division of Infection, Immunity and Respiratory Medicine Faculty of Biology, Medicine and Health Manchester Institute of Biotechnology University of Manchester Manchester UK; ^5^ Trinity Biomedical Sciences Institute Trinity College Trinity Centre for Biomedical Engineering Dublin Ireland; ^6^ Department of Mechanical, Manufacturing, and Biomedical Engineering School of Engineering Trinity College Dublin Ireland; ^7^ Trinity College Dublin & RCSI Advanced Materials and Bioengineering Research Centre Dublin Ireland

**Keywords:** bone, epigenetics, extracellular vesicles, histone deacetylase, microRNAs, tissue engineering, trichostatin A

## Abstract

Extracellular vesicles (EVs) are emerging in tissue engineering as promising acellular tools, circumventing many of the limitations associated with cell‐based therapies. Epigenetic regulation through histone deacetylase (HDAC) inhibition has been shown to increase differentiation capacity. Therefore, this study aimed to investigate the potential of augmenting osteoblast epigenetic functionality using the HDAC inhibitor Trichostatin A (TSA) to enhance the therapeutic efficacy of osteoblast‐derived EVs for bone regeneration. TSA was found to substantially alter osteoblast epigenetic function through reduced HDAC activity and increased histone acetylation. Treatment with TSA also significantly enhanced osteoblast alkaline phosphatase activity (1.35‐fold), collagen production (2.8‐fold) and calcium deposition (1.55‐fold) during osteogenic culture (*P* ≤ 0.001). EVs derived from TSA‐treated osteoblasts (TSA‐EVs) exhibited reduced particle size (1‐05‐fold) (*P* > 0.05), concentration (1.4‐fold) (*P* > 0.05) and protein content (1.16‐fold) (*P* ≤ 0.001) when compared to untreated EVs. TSA‐EVs significantly enhanced the proliferation (1.13‐fold) and migration (1.3‐fold) of human bone marrow stem cells (hBMSCs) when compared to untreated EVs (*P* ≤ 0.05). Moreover, TSA‐EVs upregulated hBMSCs osteoblast‐related gene and protein expression (ALP, Col1a, BSP1 and OCN) when compared to cells cultured with untreated EVs. Importantly, TSA‐EVs elicited a time‐dose dependent increase in hBMSCs extracellular matrix mineralisation. MicroRNA profiling revealed a set of differentially expressed microRNAs from TSA‐EVs, which were osteogenic‐related. Target prediction demonstrated these microRNAs were involved in regulating pathways such as ‘endocytosis’ and ‘Wnt signalling pathway’. Moreover, proteomics analysis identified the enrichment of proteins involved in transcriptional regulation within TSA‐EVs. Taken together, our findings suggest that altering osteoblasts’ epigenome accelerates their mineralisation and promotes the osteoinductive potency of secreted EVs partly due to the delivery of pro‐osteogenic microRNAs and transcriptional regulating proteins. As such, for the first time we demonstrate the potential to harness epigenetic regulation as a novel engineering approach to enhance EVs therapeutic efficacy for bone repair.

## Graphical abstract

Epigenetic regulation promotes the osteogenic potency of osteoblast‐derived EVs for bone repair. The HDAC inhibitor Trichostatin A (TSA) altered osteoblast epigenetic functionality through hyperacetylation, enhancing its mineralisation capacity. EVs derived from TSA treated osteoblasts (TSA‐EVs) were enriched with pro‐osteogenic microRNAs and transcriptional regulating proteins. TSA‐EVs significantly promoted hBMSCs osteogenic differentiation and mineralisation. Epigenetic reprogramming provides a novel engineering approach to enhance EVs therapeutic efficacy as an acellular tool for bone regeneration.

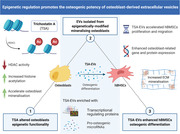

## 1 INTRODUCTION

There is a tremendous need for bone tissue due to numerous clinical situations (Baroli, 2009; Dimitriou et al., 2011), with currently 10 million people in the UK affected by musculoskeletal disorders costing the National Health Service £4.76 billion annually (Chance‐Larsen et al., 2019). Alarmingly this is anticipated to increase further in the future as a result of the growing ageing population and demand for continued quality of life in the older years. Autografts are the current gold standard treatment for the repair of critical‐sized bone defects, however, they are associated with several concerns such as their limited availability and donor site morbidity (Calori et al., 2011; Djouad et al., 2012). Consequently, there is a critical calling for new approaches to regenerate damaged bone. Hence, extensive research has been conducted within the tissue engineering field to meet the rising demand for clinically‐relevant bone tissue. Although promising cell‐based therapies have been reported (Amini et al., 2012), there has been limited clinical success due to issues associated with intensive cost, scalable manufacture of cells and ethical considerations (Izadpanah et al., 2006). Thus, there is a growing demand to develop acellular approaches for bone repair (Burdick et al., 2013).

Cells are known to secrete a range of bioactive products into the surrounding microenvironment, which have trophic effects on neighbouring cells stimulating numerous biological processes (Driscoll & Patel, 2019; Sun et al., 2019). One of these cell‐secreted factors, extracellular vesicles (EVs), have been acquiring growing interest in recent years as an acellular tool for regenerative medicine (El Andaloussi et al., 2013). EVs are cell‐derived nanoparticles, which contain a diverse biological cargo including proteins, nucleic acids and bioactive molecules, and are heavily involved in intercellular communication, regulating tissue development and homeostasis (Man et al., 2020; Raposo & Stoorvogel, 2013). The beneficial effects once attributed to cells, are now thought to be partially due to the paracrine factors delivered by EVs (Gnecchi et al., 2005; Xin et al., 2014). Hence, there has been intensive investigations into the role these nanoparticles may play as novel acellular tools for bone regeneration (Chen et al., 2019; Qin et al., 2016), overcoming the tremendous regulatory hurdles associated with the clinical translation of cell‐based therapies (Heathman et al., 2015).

In recent years, several studies have reported the considerable utility of EVs to stimulate osteogenesis (Eichholz et al., 2020; Tan et al., 2020). For example, Davies et al. demonstrated the osteoinductive capacity of EVs derived from osteoblasts, eliciting enhanced stem cell mineralisation compared to that of the current gold standard growth factor, bone morphogenic protein 2 (BMP2) (Davies et al., 2017). These osteoblast‐derived EVs may act as extracellular sites of mineral nucleation, due to their enrichment with the calcium‐channelling protein Annexin. Moreover, the role of EVs in facilitating microRNA transfer to recipient cells and promoting their osteogenic differentiation has also been demonstrated (Cui et al., 2016; Tang et al., 2017). Hence, EVs play a critical and multifunctional role in regulating osteogenesis, which is dependent on their biological cargo. Although the potential utility of these nanoparticles have been reported, numerous studies have investigated EV engineering approaches to further promote the therapeutic efficacy of these vesicles beyond their native function (Kim et al., 2020; Man et al., 2020). Of the numerous EV engineering strategies explored in the literature (Man et al., 2020), genetic modification of the EV parental cell has been extensively studied with promising results observed (Kang et al., 2015; Tao et al., 2017). However, there are issues associated with the use of this technology including high associated costs, ineffectual EV loading and increased risk of tumourigenesis (Hanna et al., 2016; Kooijmans et al., 2016). Consequently, there is a tremendous need for alternative methods in augmenting the parental cell phenotype to enhance the therapeutic viability of EVs for bone regeneration.

Altering the cells’ epigenetics via post‐translational modifications has gained increasing attention in regenerative medicine (Dompe et al., 2020; Wijnen & Westendorf, 2019). Epigenetic regulation involves controlling the transcriptional activity of the genome, without altering the underlying nucleotide sequence (Collas et al., 2008; Huynh et al., 2017), therefore providing an alternative safer method of improving EVs therapeutic efficacy through parental cell modification. Histone proteins play a critical role in regulating the structure of the chromatin. Modifying the acetylation state of the histone via augmenting the activity of histone deacetylase (HDAC) and histone acetyltransferase (HAT) have been shown to modify cell transcriptional activity (Lawlor & Yang, 2019). Hyperacetylation of the chromatin induced by the inhibition of HDAC enzymes has been demonstrated to promote osteogenic differentiation, through enhanced osteoblast‐related gene activation (Jonason et al., 2009; Man et al., 2021; Paino et al., 2014). Additionally, hyperacetylation of non‐histone proteins have also been reported to stimulate osteogenesis through transcription factor activation. Jeon et al. reported that BMP2 increased HAT acetylation of the osteogenic transcription factor Runx2 in HEK293 and C2C12 cells, inhibiting Smad specific E3 ubiquitin‐protein ligase 1 (Smurf1)‐mediated degradation of Runx2, ultimately enhancing its stability and transcriptional activity (Jeon et al., 2006). Moreover, several studies have reported the role of HDAC enzymes in binding to Runx2, T‐cell factor, nuclear factor of activated T cells and zinc finger protein 521, which results in modulation of osteogenic differentiation by silencing the expression of key osteogenic‐related genes (Choo et al., 2009; Jensen et al., 2010). HDAC inhibitors (HDACis) are small molecular compounds that have been extensive explored to reprogram the epigenome for cancer therapeutics and regenerative medicine applications (Bolden et al., 2013; Eckschlager et al., 2017; Marks, 2010). Trichostatin A (TSA) is a naturally‐derived hydroxamic acid‐based HDACi that targets class I and II HDAC isoforms (Ma et al., 2015). Several studies have demonstrated TSAs’ efficacy in stimulating both stem/progenitor cells osteogenic differentiation through hyperacetylation induced chromatin remodelling and osteogenic transcription factor activation (Jin et al., 2013; Schroeder & Westendorf, 2005; Schroeder et al., 2004).

**FIGURE 1:**
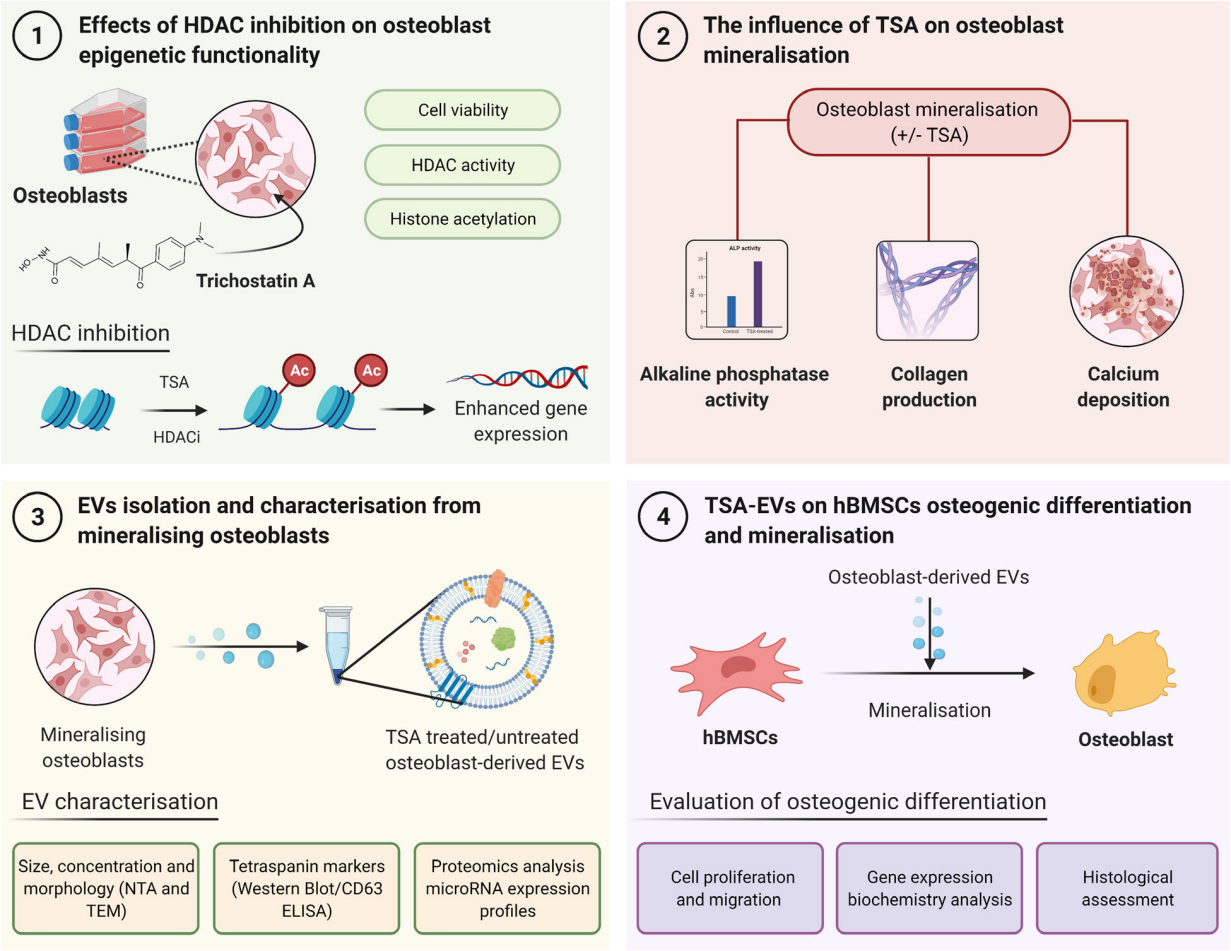
Experimental outline investigating the effects of altering osteoblast epigenetic functionality on the therapeutic potency of their EVs for bone regeneration. 1) The influence of TSA on osteoblast epigenetic functionality was assessed. 2) The effects of TSA on osteoblast mineralisation was evaluated by quantifying ALP activity, collagen production and calcium deposition. 3) EVs were isolated from TSA‐treated and untreated mineralising osteoblast over a 2‐week period, and the nanoparticles were characterised by their size, morphology, protein and microRNA expression. 4) Investigating the effects of TSA‐EV treatment on hBMSCs osteogenic differentiation. Figure created with BioRender.com

Therefore, in this present study, we investigated augmenting the epigenetic functionality of mineralising osteoblasts using the HDACi TSA to promote the therapeutic potency of osteoblast‐derived EVs for bone regeneration. We determined that 5 nM TSA effectively altered osteoblast epigenetic function and promoted its mineralising capacity. EVs isolated from TSA‐treated (TSA‐EVs) and untreated mineralising osteoblasts (MO‐EVs) were administered to human bone marrow‐derived mesenchymal stem cells (hBMSCs) to determine their efficacy in promoting osteogenic differentiation. Additionally, microRNA profiling and proteomics analysis of EVs was performed to elucidate the mechanisms in which TSA‐EVs impart their pro‐osteogenic effects (Figure 1).

## 2 MATERIALS AND METHODS

### 2.1 Cell culture and reagents

MC3T3 murine pre‐osteoblasts were purchased from American Type Culture Collection (ATCC, UK) and hBMSCs were acquired from Lonza (Lonza, UK). Basal culture media consisted of minimal essential medium (α‐MEM; Sigma‐Aldrich, UK) supplemented with 10% foetal bovine serum (FBS), 1% penicillin/streptomycin (Sigma‐Aldrich, UK) and L‐glutamine (Sigma‐Aldrich, UK). hBMSCs were used at passage 4. Mineralisation medium comprised of basal culture media supplemented with 10 mM β‐glycerophosphate (Sigma‐Aldrich, UK) and 50 µg/ml L‐ascorbic acid (Sigma‐Aldrich, UK). The synthetic glucocorticoid dexamethasone was excluded due to side effects observed in vivo (Li et al., 2005; Woolf, 2007; Xu et al., 2019) and to standardise EV isolation/dosing protocols as previously reported (Davies et al., 2017). Culture medium utilised for EV isolation and dosing was depleted of FBS‐derived EVs by ultracentrifugation at 120,000 g for 70 min prior to use.

### 2.2 Cell viability and morphology assessment

Osteoblasts were seeded at 3 × 10^3^ cells/cm^2^ within a 96‐well plate with basal medium and incubated for 24 h. Media was replaced with fresh basal medium supplemented with/without TSA (Sigma‐Aldrich, UK) (5, 10, 20, 50, 100 nM) and incubated for 1, 3 and 7 days. At each time point, AlamarBlue reagent (Thermo Scientific, UK) was added and incubated for 4 h at 37°C. Fluorescence readings was acquired using a SPARK spectrophotometer (TECAN, CH) at an excitation/emission wavelength of 540/590 nm, respectively. Employing the same protocol, the osteoblast morphology via calcein‐AM staining (Sigma‐Aldrich, UK) after 3 days of culture, observed under an EVOS fluorescent inverted microscope (Thermo Scientific, UK).

### 2.3 HDAC activity and H3K9 histone acetylation

Cells were cultured in 96‐well plates (3 × 10^3^ cells/cm^2^) in basal medium for 24 h. Medium was replaced with fresh basal medium supplemented with/without TSA (5, 10, 20, 50, 100 nM). At day 3 and 7, the HDAC activity was quantified using an *in situ* HDAC activity fluorometric assay kit (BioVision, UK) according to the manufacturer's instructions. Briefly, media was replaced with 100 µl of reaction mix and incubated for 3 h at 37°C. 100 µl of lysine developer was added then further incubated for 30 min at 37°C. Fluorescence was measured in a SPARK spectrophotometer at an excitation/emission wavelength of 368/442 nm. HDAC activity was normalised with DNA content.

Detection of H3K9 acetylation was performed using the EpiQuik™ *In Situ* Histone H3‐K9 Acetylation Assay Kit (Epigentek, USA) according to the manufacturer's protocol. The absorbance was read in a SPARK spectrophotometer at 450 nm. Histone acetylation was normalised with DNA content.

DNA quantification was determined by Quant‐iT PicoGreen DNA assay (Invitrogen, Life Technologies, UK). Briefly, cells were lysed following three freeze‐thaw cycles in 0.1% Triton™ X‐100 in Phosphate buffered saline (PBS, Lonza, UK). 90 µl of TE (10 mM Tris‐HCl, 1 mM EDTA) buffer was added to 10 µl of cell lysate in a 96‐well plate (Corning, UK). 100 µl of PicoGreen reagent was added to all samples and then incubated at 37°C for 5 min. The fluorescence was then measured in a SPARK spectrophotometer at an excitation/emission wavelength of 480/520 nm.

### 2.4 Osteoblast mineralisation with TSA

Osteoblasts were cultured in 24‐well plates at a density of 21 × 10^3^ cells/cm^2^ in basal medium for 24 h. The media was replaced with mineralisation medium supplemented with TSA for 21 days. Medium changes and TSA replenishment was performed every 48 h. Cells cultured in mineralising medium alone were used as control.

### 2.5 EVs isolation and characterisation

#### 2.5.1 EV isolation

Osteoblasts were cultured at scale in T175 culture flasks (Sarstedt, UK) and medium isolated every two days. Cells were cultured in osteogenic medium supplemented with/without 5 nM TSA for 14 days. EVs were isolated from conditioned medium (400 ml) by differential centrifugation: 2000 g for 20 min, 10,000 g for 30 min and 120,000 g for 70 min to pellet EVs (Davies et al., 2017). The supernatant was removed, and the pellet was washed in sterile PBS and centrifuged at 120,000 g for 70 min and the resultant pellet was re‐suspended in 200 µl PBS. All ultracentrifugation steps were performed utilising the Sorvall WX Ultra Series Ultracentrifuge (Thermo Scientific, UK) and a Fiberlite, F50L‐8×39 fixed angle rotor (Piramoon Technologies Inc., USA). EV characterisation was conducted following guidelines published in the Minimal Information for Studies of Extracellular Vesicles 2018 (Théry et al., 2018).

#### 2.5.2 Particle size and concentration analysis

Total EV protein concentration was determined using the Pierce BCA protein assay kit (Thermo Scientific, UK). Dynamic Light Scattering (Zetasizer Nano ZS, Malvern Instruments, UK) was used to analyse polydispersity index (PDI). Nanoparticle tracking analysis was performed on EV samples to determine particle size and concentration using a ZetaView® instrument (Particle Metrix, Germany). EV samples were diluted 1:100 in PBS and injected into the ZetaView®, where 4 × 40 s videos were obtained of particles in motion. Particle size and concentration was determined with the ZetaView® software. The quantity of CD63 positive particles was confirmed using the ExoELISA‐ULTRA Complete Kit (CD63 Detection) (System Biosciences, USA) following the manufacturers’ protocol.

#### 2.5.3 Transmission electron microscopy (TEM)

EV imaging was conducted via a JEOL JEM1400 transmission electron microscope (TEM) coupled with an AMT XR80 digital acquisition system. Samples were physisorbed to 200 mesh carbon‐coated copper formvar grids (Agar Scientific, UK) and negatively stained with 1% uranyl acetate.

#### 2.5.4 Immunoblotting

Immunoblotting analysis was used to confirm the presence of EV as previously described (Nikravesh et al., 2019). Briefly, following the electrophoretic separation of proteins using precast gels (4%‐15% Mini‐PROTEAN TBX, Biorad, UK), gels were blotted on polyvinylindene disluoride membranes (Fisher Scientific, UK) and blocked with EveryBlot blocking buffer (BioRad, UK). The blots were incubated overnight at 4°C with primary antibodies to Alix (1:1000 dilution, Santa Cruz, USA), Annexin 2 (1:2000 dilution, Abcam, UK), CD9 (1:1000 dilution, Abcam, UK) and calnexin (1:1000 dilution, Abcam, UK). The membranes were incubated with the appropriate secondary antibody, anti‐rabbit for Annexin 2, CD9 and calnexin (1:3000 dilution, Cell Signaling, UK), and anti‐mouse for Alix (1:3000 dilution, Cell Signaling, UK), for 1 h at room temperature. Chemiluminescence detection of bands were images with ChemiDoc XRS+ system (BioRad, UK) by a chemiluminescence reaction using Clarity™ Western ECL substrate (BioRad, UK) and Image Lab software (Life Science Research, BioRad, UK) following supplier's instructions.

### 2.6 EVs microRNA isolation, analysis and bioinformatics

Total EV RNA was isolated using the Qiagen miRNeasy Mini Kit (Qiagen, UK) according to the manufacturers’ protocol and RNA quantity/purity assessed by measuring 260/280 nm absorbance ratio using a NanoQuant plate and a SPARK spectrophotometer. RNA content was normalised with particle number. Global expression patterns of EV miRNAs were examined by using a microarray chip containing 1963 probes for murine microRNAs (miR‐Base 22). Microarray analysis of EV microRNAs (500 ng of total RNA per EV sample) was performed by LC Sciences (Houston, USA) using the μParaflo™ microRNA microarray biochip technology. Differentially expressed microRNAs were defined by a threshold of *P* < 0.05 and fold change > 2.0. Significantly altered microRNAs were further analysed to predict their target genes and pathways. Target genes of miRNAs were predicted using two algorithms, DIANA‐Tarbase v 7.0 and DIANA‐microT‐CDS. A microT threshold of 0.8 when microT‐CDS were used for target gene prediction. DIANA‐mirPath v.3 was utilised to perform hierarchical clustering of miRNAs, to assess gene ontology (GO) annotation and Kyoto Encyclopaedia of Genes and Genomes (KEGG) pathways based on interaction level using experimentally validated miRNA interactions derived from DIANA‐Tarbase v7.0 and/or predicted miRNA gene targets provided by DIANA‐microT‐CDS (Vlachos et al., 2012). Benjamini and Hochberg's false discovery rate (FDR) was applied with the significant threshold set at a p‐value of < 0.05.

### 2.7 Sample preparation for proteomics analysis

Protein extraction for proteomic analysis was performed by adding 400 µl acetone (Thermo Scientific, UK) to 100 µl of EVs previously isolated and resuspended in PBS. Samples were vortexed and incubated at ‐80°C for 1 h. After incubation, the samples were centrifuged at 14,000 g for 10 min. The supernatant was discarded, the pellet dried by inverting and then resuspended in 0.1 M ammonium bicarbonate (Acros Organics, USA), 0.1% RapiGest (Waters Corpo., USA) in LC‐MS grade water (Thermo Scientific, UK) to a final concentration of 1 µg/µl.

Proteins were denatured with 1.5 µl of 1% (w/v) RapiGest in 50 mM ammonium bicarbonate and incubated at 80°C for 45 min. Following incubation, 100 mM DTT (1 µl) was added and incubated for a further 30 min at 60°C to reduce the proteins, before being alkylated with 200 mM iodoacetamide (1 µl) at room temperature for 30 min. Trypsin 1:50 (w/w) (Gold Mass Spectrometry grade, Promega, USA) was added to each sample for proteolytic digestion and left to incubate overnight at 37°C. Trifluoroacetic acid was added to a final concentration of 0.5% (v/v) to hydrolyse the RapiGest and heated for a further 45 min at 37°C, before centrifuging for 25 min at 18,000 g. The supernatant was collected and 5 µl aliquoted for LC‐MS analysis. Aliquoted samples were diluted 1:4 (v/v) with 15 µl of 0.1% formic acid (v/v) to provide a working solution of 200 ng/µl.

### 2.8 LC‐MS analysis

Extracted peptides obtained from the isolated vesicles were analysed by one dimensional nanoscale reversed‐phase (RP) chromatography using an ACQUITY M‐Class UPLC (Waters Corp., USA) configured for trap and elute. Peptides were loaded (1 µl injection, 200 ng on‐column) onto a Symmetry C18 5 µm, 2 cm × 180 µm pre‐column (Waters Corp., USA) with aqueous 0.1% (v/v) formic acid using a flow rate of 15 µl/min for 2 min. Mobile phases consisted of water with 0.1% (v/v) formic acid (mobile phase A) and acetonitrile with 0.1% (v/v) formic acid (mobile phase B). Peptides were eluted from the pre‐column and separated over a 90 min gradient using a HSS T3 C18 1.7 µm, 15 cm × 75 µm analytical column (Waters Corp., USA). The gradient consisted of 3 ‐ 40% mobile phase B over 60 min at a flow rate of 400 nl/min, whilst maintaining the analytical column temperature at 35°C. Lock mass consisting of [Glu1]‐Fibrinopeptide was delivered to the reference sprayer of the MS source using the M‐Class Auxillary Solvent Manager with a flow rate of 1 µl/min.

MS data were collected on a Synapt XS mass spectrometer (Waters Corp., UK) operated in positive electrospray ionisation (ESI) mode with a nominal resolution of 25,000 FWHM (V optics). The capillary voltage was 3.2 kV, cone voltage was 35 V and source temperature was set at 100°C. Data were acquired over 50 ‐ 2000 Da mass range with a scan time of 0.5 s. All mass spectral data were acquired in continuum mode using UDMSE to obtain fragmentation data simultaneously (Distler et al., 2016; Rodriguez‐Suarez et al., 2013). Function one (low energy) data were collected using a constant trap and transfer energy of 6 eV whilst the second (high energy) function consisted of a transfer collision energy ramp of 19 to 45 eV. For mass accuracy, [Glu1]‐fibrinopeptide (m/z = 785.8426) was acquired as lock mass at a concentration of 100 fmol/µl (in 50:50 CH_3_CN/H_2_O, 0.1% formic acid). Lock mass scans were collected every 60 s and averaged over 3 scans to perform mass correction. The time‐of‐flight mass analyser was externally calibrated over the acquisition mass range (50 ‐ 2000 Da) before analysis with a NaCsI mixture (Waters API MS Calibration Solution, 2 µg/µl sodium iodide: 50 ng/µl cesium iodide in 50:50 isopropanol:water, Waters Corp., USA). These data were collected using MassLynx v 4.1 software (Waters Corp., UK) in a randomized order with three technical replicates acquired per sample.

### 2.9 LC‐MS data analysis

Progenesis QI for Proteomics (Nonlinear Dynamics, UK) was used to process all data. Retention time alignment, peak picking and normalization were conducted to produce peak intensities for retention time (RT) and m/z data pairs. Data were searched against reviewed entries of a Mus musculus UniProt database (17,048 reviewed entries, release 2020_05) to provide protein identifications with a FDR of 1%. A decoy database was generated as previously described (Li et al., 2009) allowing for protein/peptide identification rates to be determined. Peptide and fragment ion tolerances were determined automatically, and searches allowed for one missed cleavage site. Carbamidomethyl of cysteines was applied as a fixed modification, whilst oxidation of methionine and deamidation of asparagine/glutamine were set as variable modifications.

### 2.10 EV treatment of hBMSCs

#### 2.10.1 EV labelling

EVs were labelled using Cell Mask™ Deep Red Plasma Membrane Stain (1:1000 in PBS) (Thermo Scientific, UK) and incubated for 10 min. Labelled EVs were washed twice with PBS via ultracentrifugation at 120,000 g for 70 min. Cells were seeded at a density of 4 × 10^3^ cells/cm^2^ in a chamber slide (Corning, UK) for 24 h. Media was replaced with fresh basal media supplemented with labelled EVs. After 2, 6 and 24 h, cells were fixed with 10% (v/v) neutral buffered formalin (NBF, Cellpath, UK), stained with Alexa Fluor 488 phalloidin (1:20) (Cell Signalling Technology, UK) and then mounted with Prolong ^™^ Gold Antifade Mountant with DAPI (Thermo Scientific, UK) to label the actin cytoskeleton and nuclei respectively. Treated cells were imaged with the aforementioned EVOS fluorescent inverted microscope.

#### 2.10.2 hBMSCs proliferation and migration

The influence of EVs on hBMSCs proliferation was assessed via quantification of DNA content. Briefly, cells were seeded at 1 × 10^4^ cells/cm^2^ in basal media for 24 h. Then, media was replaced with fresh basal medium supplemented with TSA‐EVs or MO‐EVs (10 µg/ml). Cells cultured in basal medium alone were used as a control. DNA content was evaluated on day 3, 7 and 14 utilising the previously described DNA quantification assay. Migration rate was measured by performing scratch assays. Briefly, cells at a density of 30 × 10^3^ cells/cm^2^ in a 6‐well plate were seeded and allowed to adhere for 24 h. A scratch was applied with a 200 µl pipette tip and the width was measured as the baseline. Cell were incubated with basal medium with/without EVs (10 µg/ml) for 3 days. Cells cultured in basal medium alone was used as the control. The rate of migration was assessed under a light microscope (EVOS XL Core, Invitrogen, UK).

#### 2.10.3 hBMSC osteogenic culture

hBMSCs were seeded in 24‐well plates (Nunc, UK) at a density of 21 × 10^3^ cells/cm^2^ in basal medium and incubated for 24 h. The media was replaced with mineralisation medium supplemented with TSA‐EVs or MO‐EVs (10 µg/ml) for 28 days unless stated otherwise. EV‐supplemented mineralisation medium changes were performed every 48 h. Cells cultured in mineralising medium alone was used as the untreated control.

### 2.11 Quantitative RT‐qPCR analysis

RNase mini kit (Qiagen, UK) was used to extract total RNA from EV treated and untreated hBMSCs according to the manufacturer's protocol. Commercially available primers (Supplementary Table 1) (Primerdesign, UK) were used to quantify levels of alkaline phosphatase (*ALP*), collagen type I (*COL1A*), bone sialoprotein (*BSP*) and osteocalcin (*OCN*). Glyceraldehyde 3‐phosphate dehydrogenase (*GAPDH*) was used as the internal reference for mRNA. Isolated RNA was amplified in a 20 µl reaction with a 96‐well PCR plate (Starlab, UK). Amplification occurred using the AriaMx Real‐Time PCR System (Agilent Technologies, UK). For each sample, the cycle threshold (Ct) value was acquired and the comparative Ct method (2^–∆∆Ct^) was utilised to quantify the gene expression levels relative to the housekeeping gene.

### 2.12 Alkaline phosphatase activity

ALP activity was determined using the 4‐nitrophenyl colourimetric phosphate liquid assay (pNPP, Sigma‐Aldrich, UK). 10 µl of cell lysate was added to 90 µl of pNPP and incubated for 60 min at 37°C. The absorbance at 405 nm was read on a SPARK spectrophotometer. ALP activity was normalised with DNA content.

### 2.13 In‐Cell Western assay

Intracellular protein expression was assessed with the In‐Cell Western assay (ICW) as previously reported (Man et al., 2021). Briefly, cells were fixed in 10% NBF, permeabilised by 0.1% Triton™ X‐100 in PBS and non‐specific binding was blocked using the Odyssey blocking buffer (Li‐Cor Biosciences, UK). Samples were incubated overnight at 4°C with primary antibodies to ALP (1:300), Col1a (1:200), and OCN (1:400) (Abcam, UK) in Odyssey® buffer. Cells were then incubated with IRDye 800CW secondary antibody (1:800) and CellTag™ 700 stain (1:500; Li‐Cor Biosciences, UK) in Odyssey blocking buffer for 1 h. Prior to scanning, samples were then washed in 0.1% Tween20 in PBS and analysed on an Odyssey SA Imaging System (Li‐Cor Biosciences, UK) at 700 and 800 nm. Quantitative analysis was performed using the Image Studio (Li‐Cor Biosciences: version 5).

### 2.14 Collagen production

Extracellular matrix collagen deposition was evaluated with picrosirius red staining. Briefly, cells were washed twice in PBS and fixed in 10% NBF for 30 min, prior to staining with 0.1% sirius red in saturated picric acid (Sigma‐Aldrich, UK) for 1 h. The unbound dye was removed by washing in 0.5 M acetic acid followed by distilled water wash and left to air dry prior to imaging using light microscopy (EVOS XL Core, Invitrogen, UK). To quantify collagen staining, 0.5 M sodium hydroxide was used to elute the bound dye and absorbance were read at 590 nm using the SPARK spectrophotometer.

### 2.15 Mineral deposition

To evaluate mineralisation, calcium deposition was assessed via alizarin red staining. Cells were washed twice in PBS and fixed in 10% NBF for 30 min. Following fixation, cells were washed in distilled water and then incubated with alizarin red solution (Sigma‐Aldrich, UK) for 10 min. The unbound dye was removed by washing is distilled water. Staining was visualised using light microscopy (EVOS XL Core, Invitrogen, UK). For alizarin red quantification, samples were de‐stained with 10% cetylpyridinium chloride (Sigma‐Aldrich, UK) for 1 h and then absorbance were read at 550 nm using the SPARK spectrophotometer.

### 2.16 Statistical analysis

For all data presented, experiments were performed in triplicate. All statistical analysis was assessed using the IBM SPSS software (IBM Analytics, version 21). The Shapiro‐Wilk test was used to analyse the normality of data. Data that was proven to be normally distributed were analysed using parametric students' T‐test, one‐way ANOVA, or paired T‐test. Non‐normally distributed data were assessed using non‐parametric Mann‐Whitney t‐test or Kruskal‐Wallis ANOVA. P values equal to or lower than 0.05 was considered as significant. *P ≤ 0.05, **P ≤ 0.01 ***P ≤ 0.001.

## 3 RESULTS

### 3.1 The effects of TSA on osteoblast epigenetic functionality

**FIGURE 2:**
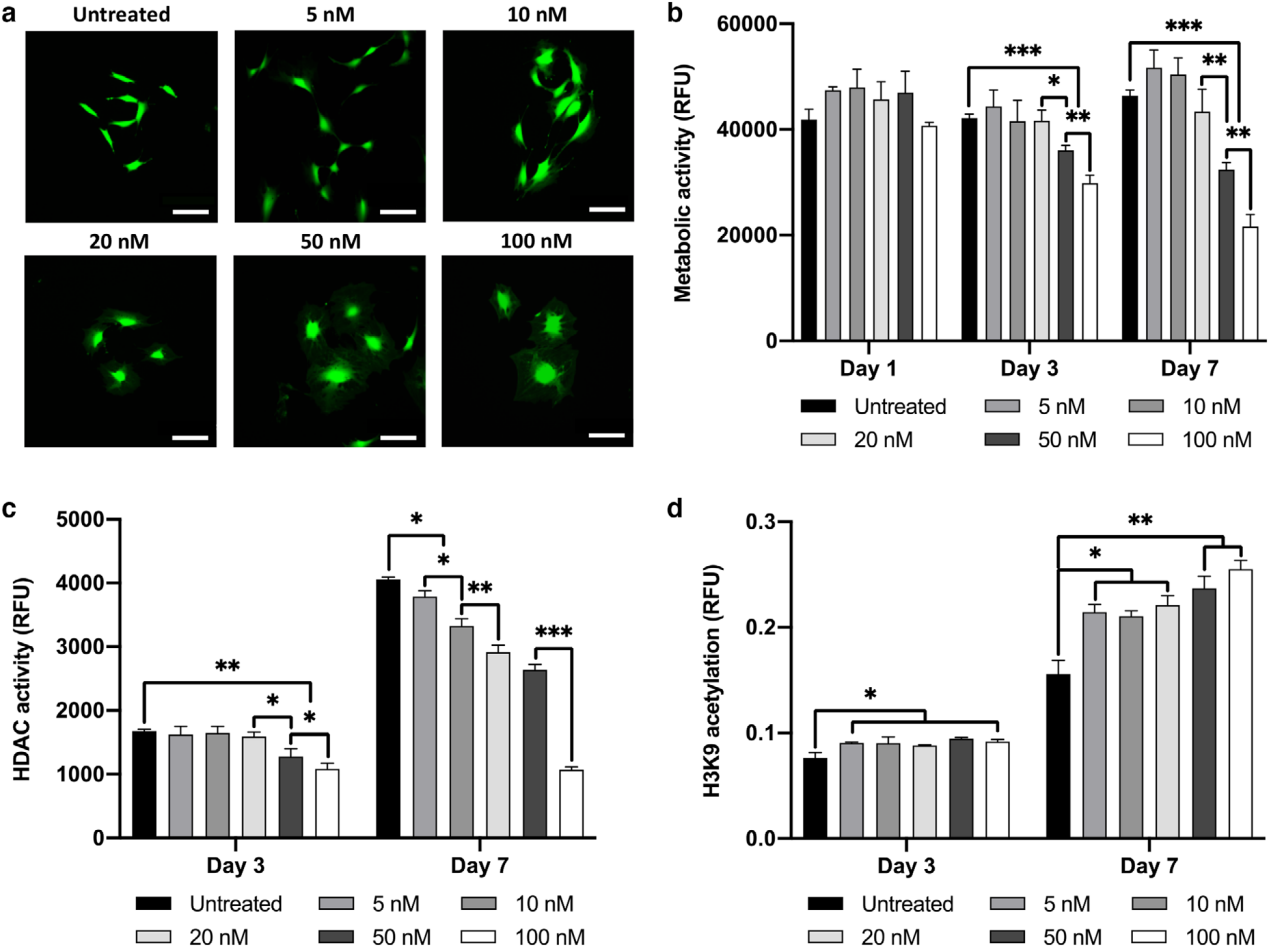
The effect of TSA on osteoblasts viability and epigenetic functionality. TSA caused a time‐dose dependant effect on osteoblast a) morphology (day 3) and b) metabolic activity. Treatment with TSA altered osteoblast c) HDAC activity and d) H3K9 histone acetylation in a time‐dose dependent manner. Data are expressed as mean ± SD (n = 3). *P ≤ 0.05, **P ≤ 0.01 and ***P ≤ 0.001. Scale bar = 100 µm

To determine the effects of TSA on osteoblast viability, cellular morphology and metabolic activity was assessed. Alterations in osteoblast morphology was observed upon increasing TSA dosages, where cells transitioned from an elongated, fibroblast‐like shape to a larger, flattened morphology (Figure 2a). A time‐dose dependent decrease in osteoblast viability was observed following TSA treatment, with concentrations of ≥ 50 nM for 3 and 7 days significantly reducing osteoblast metabolic activity compared to that in the untreated cells (*P* ≤ 0.001) (Figure 2b). This reduction in metabolic activity was observed alongside substantial variations in HDAC activity and histone acetylation levels. A time‐dose dependant decrease in HDAC activity was observed upon TSA treatment, where concentrations of ≥ 20 nM (*P* ≤ 0.05) and ≥ 5 nM (*P* ≤ 0.001) significantly reduced HDAC activity compared to that in the untreated cells at day 3 and 7 respectively (Figure 2c). Additionally, TSA elicited a time‐dose dependent increase in histone H3K9 acetylation, with concentrations of ≥ 5 nM for both day 3 and 7 significantly enhancing histone acetylation levels compared to that in the untreated cells (*P* ≤ 0.05 ‐ 0.01) (Figure 2d).

### 3.2 TSA promotes osteoblast mineralisation

**FIGURE 3:**
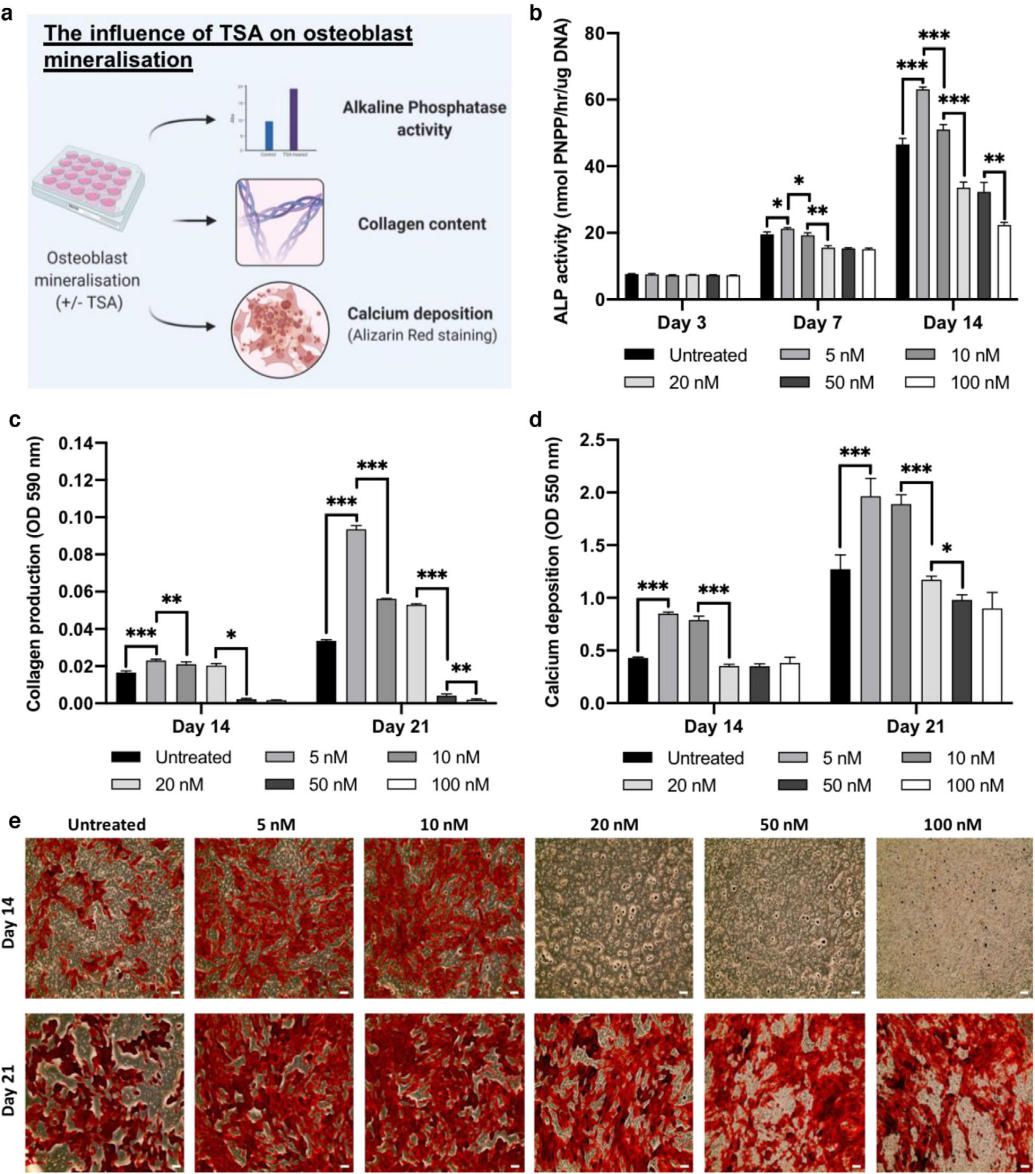
TSA treatment promoted osteoblast mineralisation. a) Schematic representation regarding the assessment of TSA on osteoblast mineralisation. Treatment with TSA elicited a time‐dose dependent effect on osteoblast b) ALP activity, c) extracellular matrix collagen production and d, e) calcium deposition. Data are expressed as mean ± SD (n = 3). *P ≤ 0.05, **P ≤ 0.01 and ***P ≤ 0.001. Scale bar = 200 µm

The influence of TSA treatment on osteoblast differentiation was evaluated by quantifying ALP activity, collagen production and calcium deposition (Figure 3a). A time‐dose dependant effect on osteoblast ALP activity was observed following TSA treatment. 5 nM TSA substantially increased ALP activity when compared to cells treated with higher TSA concentrations (≥ 10 nM) (1.1, 1.23‐fold) (*P* ≤ 0.01 ‐ 0.001) and the untreated cells (1.1, 1.35‐fold) (*P* ≤ 0.05 ‐ 0.001) at day 7 and 14 (Figure 3b). Variations in ALP activity were followed by significant changes in extracellular matrix collagen production (Figure 3c). TSA at 5 nM significantly enhanced collagen deposition when compared to the other TSA‐treated groups (≥ 10 nM) (≥ 1.1, 1.7‐fold) and the untreated control (1.4, 2.8‐fold) at both day 14 and 21, respectively. TSA treatment induced a time‐dose dependent effect on osteoblast extracellular matrix mineralisation. At day 14 and 21, 5 and 10 nM TSA significantly increased calcium deposition compared to that of the untreated cells and the ≥ 20 nM TSA treated groups (*P* ≤ 0.001) (Figure 3d, e). 5 nM TSA treatment was utilised for subsequent experiments.

### 3.3 Characterisation of EVs derived from TSA treated osteoblasts

**FIGURE 4:**
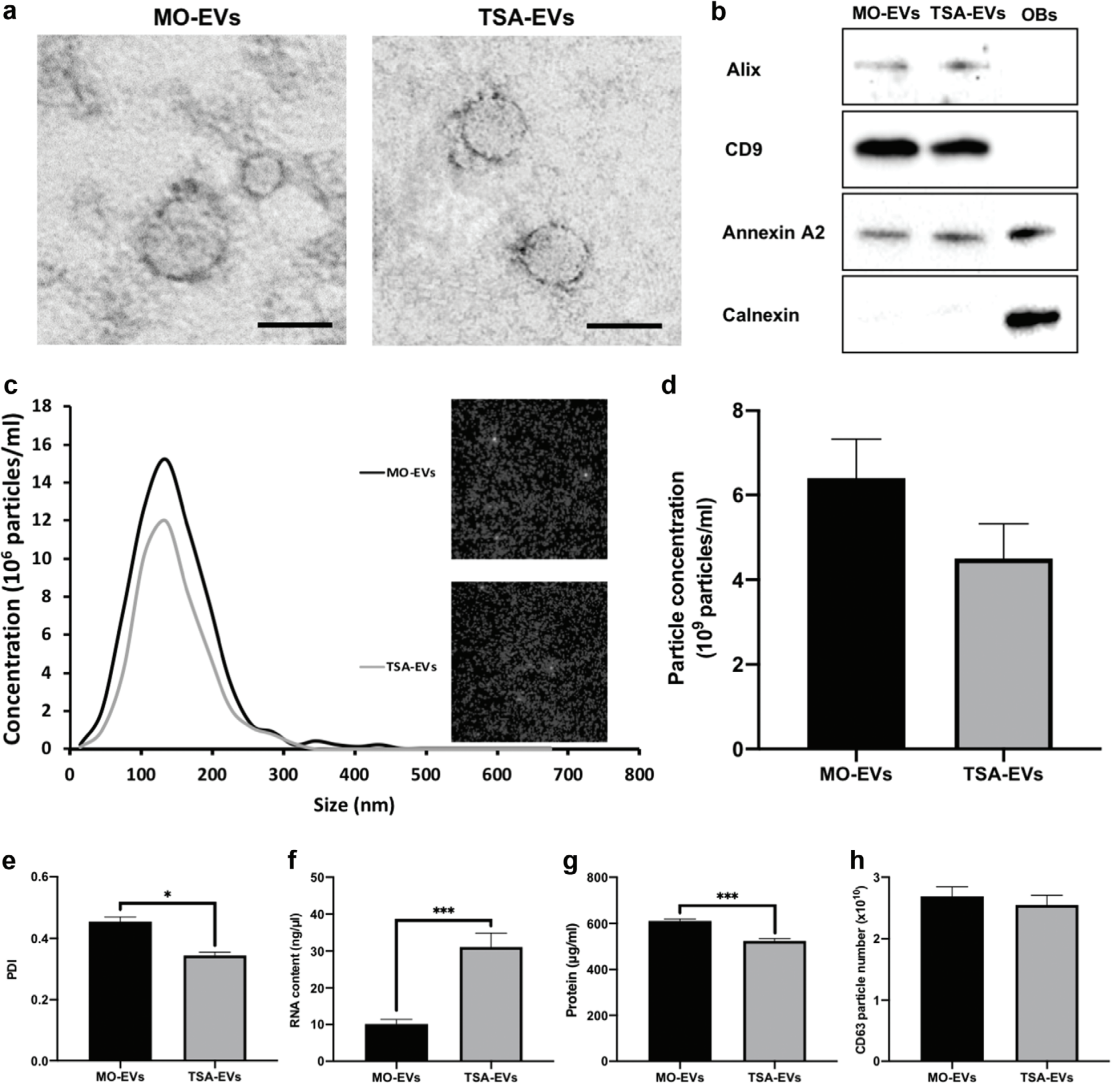
Characterisation of EVs isolated from TSA treated and untreated mineralising osteoblasts. a) TEM image of EVs isolated from TSA‐treated and untreated osteoblasts. Scale bar = 50 nm. b) Western blot analysis confirming the presence of EV markers (Alix, CD9, and Annexin A2) and absence of Calnexin (OBs ‐ osteoblast cell lysate control). c) Particle size distribution of isolated EV samples from NTA. Insert shows snapshot of particles during analysis. d) EV particle size and concentration. e) Polydispersity index of EVs. f) EVs RNA quantification. g) EVs protein content. h) CD63+ particles number. Data are expressed as mean ± SD (n = 3). *P ≤ 0.05 and ***P ≤ 0.001

EVs were isolated from untreated/TSA‐treated osteoblast conditional media over a 2‐week period via differential centrifugation. TEM imaging demonstrated the presence of particles in both groups of a typical size and spherical morphology of EVs, where these nanoparticles displayed heterogeneity in their diameters (Figure 4a and Supplementary Figure 1a). Immunoblotting confirmed the presence of Alix, CD9 and Annexin A2 proteins in both EV groups and the absence of Calnexin expression (Figure 4b). NTA analysis demonstrated that the EVs exhibited an average diameter of 136 and 129 nm for the MO‐EVs and TSA‐EVs respectively (Figure 4c, d) (*P* > 0.05). There was a 1.4‐fold reduction in the concentration of TSA‐EV particles (4.5 ± 0.82 × 10^9^/ml) compared to MO‐EVs (6.4 ± 0.92 × 10^9^/ml), although not significant (Figure 4d) (*P* > 0.05). TSA‐EVs exhibited a more monodisperse population (1.34‐fold) when compared to the MO‐EVs (PDI of 0.34 and 0.45, respectively) (Figure 4e) (*P* ≤ 0.05). Additionally, the TSA‐EVs contained substantially enhanced RNA quantity (3‐fold) when compared to that within the MO‐EVs (Figure 4f) (*P* ≤ 0.001). A significantly reduced protein content from TSA‐EVs was observed when compared to MO‐EVs (1.16‐fold) (Figure 4g) (*P* ≤ 0.001). Moreover, there was a slight non‐significant decrease (1.05‐fold) in the quantity of CD63 positive particles in the TSA‐EV group compared to MO‐EVs (Figure 4h) (*P* > 0.05).

### 3.4 TSA‐EVs promoted the proliferation, migration and osteogenic differentiation of hBMSCs

**FIGURE 5:**
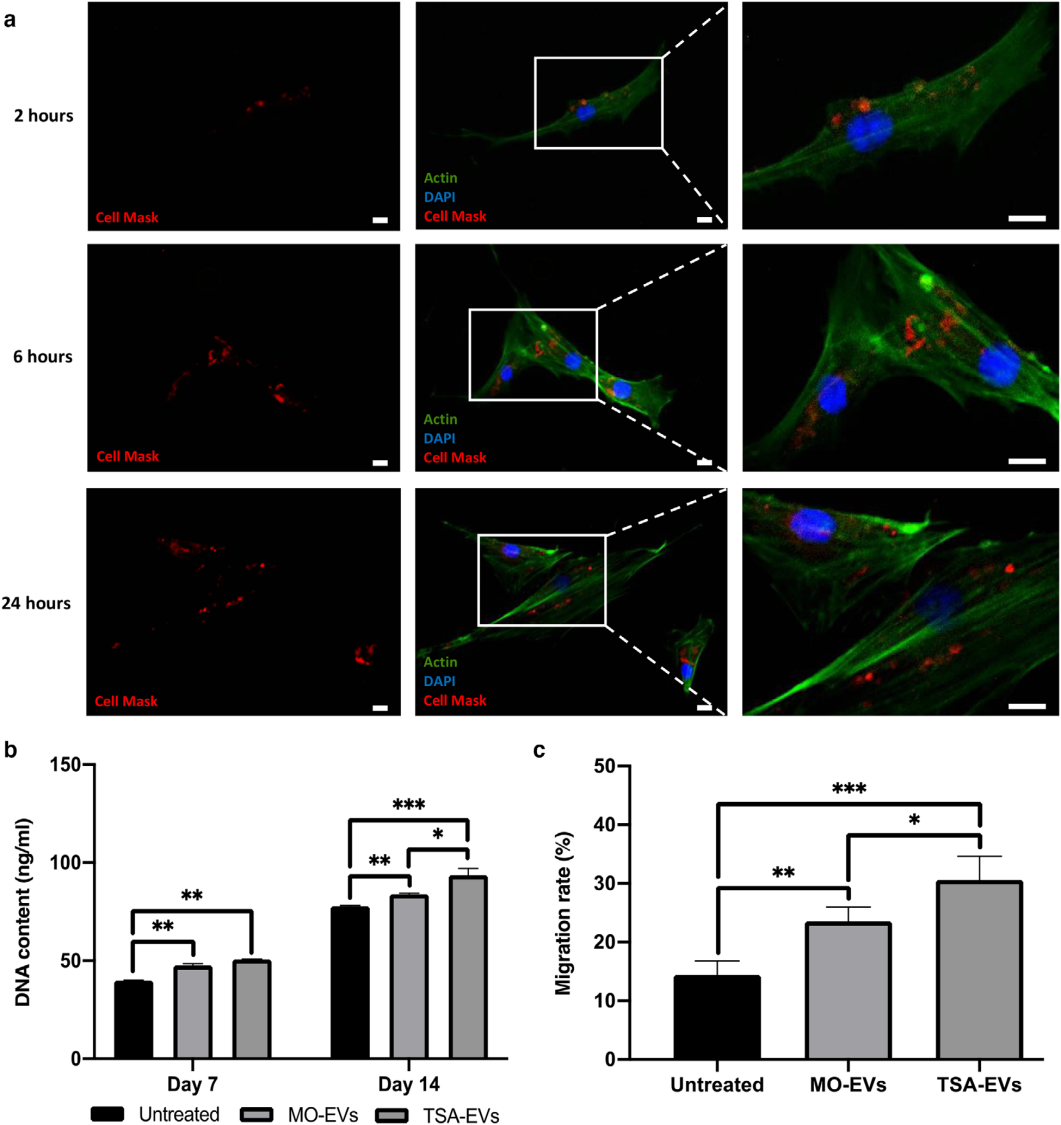
The influence of TSA‐EVs on hBMSCs general behaviour. a) Immunofluorescent images of Cell Mask labelled osteoblast‐derived EVs uptake by hBMSCs were taken at indicated times. Scale bar = 20 µm. The effects of TSA‐EVs on hBMSCs b) proliferation and c) migration. Data are expressed as mean ± SD (n = 3). *P ≤ 0.05, **P ≤ 0.01 and ***P ≤ 0.001

The influence of TSA‐EVs on hBMSCs general behaviour was initially assessed. Cell Mask labelled osteoblast‐derived EVs were successfully internalised by hBMSCs, with labelled EVs situated within the cells’ cytoplasm (Figure 5a). A time‐dependant accumulation of labelled EVs in hBMSCs was observed. Control samples are shown in Supplementary Figure 1b. Treatment with TSA‐EVs significantly increased hBMSCs proliferation in a time‐dependant manner when compared to that of the MO‐EVs treated (*P* ≤ 0.05) and untreated cells (Figure 5b) (*P* ≤ 0.01 ‐ 0.001). Additionally, hBMSCs migration was substantially enhanced following treatment with TSA‐EVs when compared to the MO‐EVs treated (1.3‐fold) (*P* ≤ 0.05) and untreated cells (2.1‐fold) (*P* ≤ 0.001) (Figure 5c).

**FIGURE 6:**
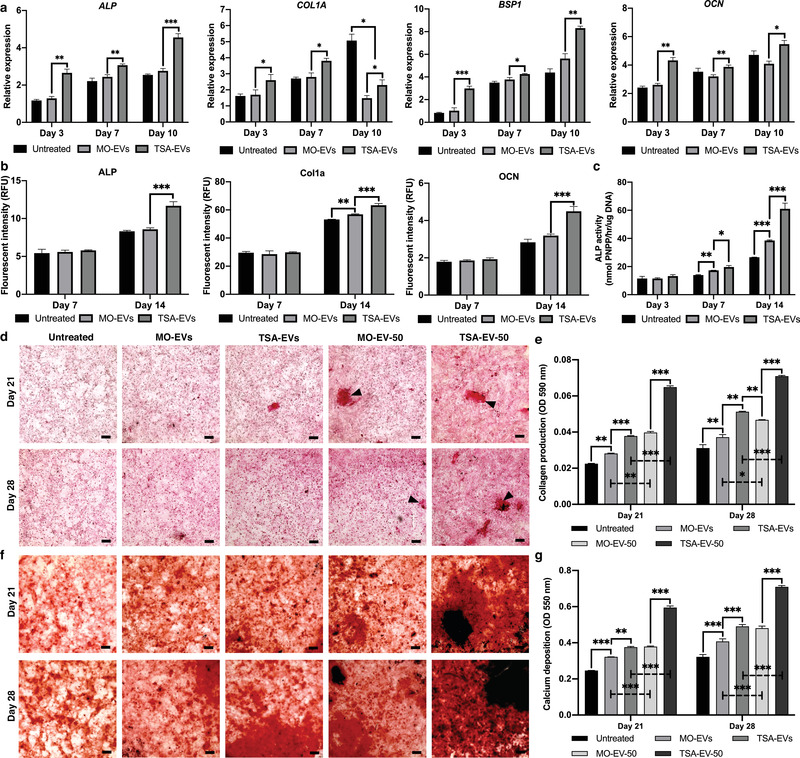
TSA‐EVs promoted hBMSCs osteogenic differentiation and mineralisation. a) Gene expression levels of *ALP, COL1A, BSP1* and *OCN* were measured in TSA‐EV, MO‐EV treated and untreated hBMSCs during osteogenic culture. b) The intracellular protein levels of ALP, Col1a and OCN in EV treated hBMSCs analysed by ICW. c) The effects of TSA‐EVs on hBMSCs ALP activity during osteogenic culture. d) Picrosirius red staining for collagen production of EV‐treated hBMSCs. Black arrows highlight collagen rich mineral nodule‐like structures. e) Quantitative analysis of picrosirius red collagen staining. f) Alizarin red staining for calcium deposition on EV‐treated hBMSCs. Black staining indicates mineral nodule formation. g) Quantitative analysis of alizarin red staining. Scale bars = 200 µm. (MO‐EV, TSA EV; 10 µg/ml) (MO‐EV‐50, TSA‐EV‐50; 50 µg/ml). Data are expressed as mean ± SD (n = 3). *P ≤ 0.05, **P ≤ 0.01 and ***P ≤ 0.001

The effects of TSA‐EV treatment on hBMSCs osteogenic differentiation was evaluated by quantifying osteoblast‐related gene expression and intracellular protein expression. MO‐EV treatment elicited a slight non‐significant increase in *ALP, COL1A, BSP1* and *OCN* gene expression (*P* > 0.05) throughout osteogenic culture, whilst displaying a decrease in OCN expression at day 7 and 10 when compared to the untreated group (*P* > 0.05). Treatment with TSA‐EVs significantly upregulated hBMSCs mRNA expression levels of *ALP, COL1A, BSP1* and *OCN* when compared to MO‐EVs treated (Figure 6a) (*P* ≤ 0.05 ‐ 0.001). *COL1A* mRNA expression for MO‐EV and TSA‐EV treated cells was significantly downregulated at day 10 when compared to untreated cells (P ≤ 0.05). Intracellular levels of osteoblast‐related proteins within hBMSCs was analysed by ICW (Figure 6b). Treatment with TSA‐EVs significantly enhanced hBMSCs intracellular protein levels of ALP (1.36, 1.4‐fold), Col1a (1.11, 1.18‐fold) and OCN (1.41, 1.58‐fold) when compared to that of the MO‐EVs treated and the untreated cells at day 14 (*P* ≤ 0.001) (Figure 6b). The MO‐EV group exhibited a significant increase in hBMSCs Col1a expression at day 14 (*P* ≤ 0.001), with a slight non‐significant enhancement in expression levels of ALP and OCN compared to the untreated control (*P* > 0.05).

The effects of TSA‐EV treatment on hBMSCs extracellular matrix mineralisation was assessed by quantifying ALP activity, collagen production and calcium deposition. ALP activity was substantially enhanced in hBMSCs treated with TSA‐EVs when compared to that of the MO‐EVs treated (1.2, 1.6‐fold) and untreated cells (1.4, 2.3‐fold) at day 7 and 14 (*P* ≤ 0.05 ‐ 0.001) (Figure 6c). Alterations in hBMSCs ALP activity were followed by significant changes in extracellular matrix collagen production. Osteoblast‐derived EV treatment elicited a time‐dose dependent increase in hBMSCs collagen deposition during osteogenic culture. The MO‐EVs (1.25, 1.26‐fold) and TSA‐EVs treated (1.68, 1.64‐fold) groups exhibited significantly increased collagen staining compared to the untreated control on day 21 and 28 (*P* ≤ 0.01 ‐ 0.001) (Figure 6d, e). The TSA‐EV treated cells displayed a 1.34‐ and 1.3‐fold enhancement in collagen deposition when compared to that of the MO‐EVs treated group on day 21 (*P* ≤ 0.001) and 28 (*P* ≤ 0.01) (Figure 6e). Moreover, 50 µg/ml EV treatment significantly increased collagen production when compared to 10 µg/ml EVs treated cells on day 21 and 28 (MO‐EV‐50 vs. MO‐EVs (1.41, 1.19‐fold)) (TSA‐EV‐50 vs. TSA‐EVs (1.72, 1.38‐fold)) (*P* ≤ 0.05 ‐ 0.001). The TSA‐EV‐50 group exhibited a 1.63‐ and 1.51‐fold increase in collagen production when compared to MO‐EV‐50 treated cells (P ≤ 0.001), with substantially increased quantity of collagen rich mineral nodule‐like structures (black arrows). Interestingly, the TSA‐EV treated cells displayed significantly enhanced collagen deposition when compared to the MO‐EV‐50 group on day 28 (1.1‐fold) (*P* ≤ 0.01). TSA‐EV treatment led to a time‐dose dependent increase in alizarin red staining for calcium deposition when compared to that of the MO‐EV treated and untreated cells, with mineral‐like nodules deposited throughout (Figure 6f). Quantitative analysis revealed that treatment with TSA‐EVs significantly increased extracellular matrix calcium deposition when compared to that of the MO‐EV treated (1.17, 1.2‐fold) (*P* ≤ 0.01 ‐ 0.001) and untreated cells (1.52, 1.52‐fold) (*P* ≤ 0.001) after 21 and 28 days of osteogenic culture (Figure 6g). Additionally, 50 µg/ml MO‐EV and TSA‐EV treatment further enhanced hBMSCs calcium deposition when compared to 10 µg/ml EVs treated cells on day 21 and 28 (MO‐EV‐50 vs. MO‐EVs (1.17, 1.18‐fold)) (TSA‐EV‐50 vs. TSA‐EV (1.58, 1.45‐fold)) (*P* ≤ 0.001). Moreover, the TSA‐EV‐50 group exhibited a significantly increased accumulation of calcium deposits when compared to the MO‐EV‐50 treated cells on day 21 and 28 (1.57, 1.47‐fold) (*P* ≤ 0.001), with enhanced quantity of mineralised nodule formations (black staining).

### 3.5 TSA induced hyperacetylation altered the microRNA profile of osteoblast‐derived EVs

**FIGURE 7:**
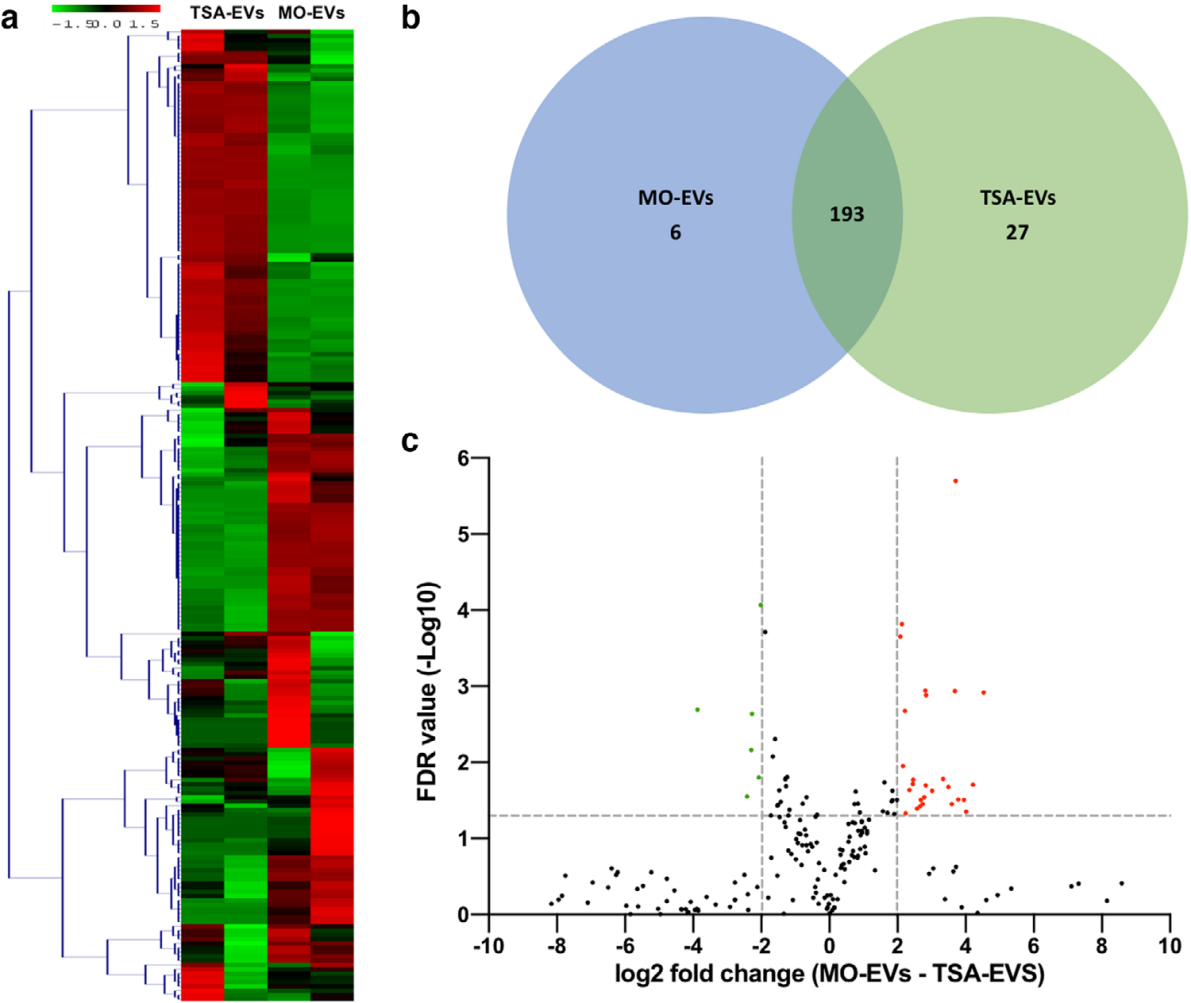
Differential expression of microRNAs derived from TSA‐EVs and MO‐EVs. a) Hierarchical clustering analysis of microRNAs that were differentially expressed between TSA‐EVs and MO‐EVs. b) Venn diagram comparing microRNAs differentially expressed from TSA‐EVs and MO‐EVs. A total of 193 shared microRNAs; 27 microRNAs upregulated in TSA‐EVs and 6 microRNAs upregulated in MO‐EVs. c) Volcano plot displaying Log_2_ values for the microRNAs fold‐change against Log_10_ FDR. MicroRNAs with a Log_2_ fold difference below 2 and a statistical value of > 0.05 were not considered to be statistically significant (vertical and horizontal lines respectively). The red points in the plot represents the significantly upregulated TSA‐EV microRNAs, the green points represent significantly upregulated MO‐EVs microRNAs

To further elucidate the influence of epigenetic modification on osteoblast‐derived EV and its possible role in the enhanced osteoinductive properties, the EVs microRNA expression was profiled. As shown in Figure 7a, hierarchical clustering revealed differential microRNAs expression in TSA‐EVs and MO‐EVs. A total of 27 microRNAs species were upregulated, while 6 were downregulated (fold change cut off, ±2.0; *P* < 0.05) in the TSA‐EVs compared to that in the MO‐EVs (Figure 7b). Volcano plots showed the variation of microRNAs expression between TSA‐EVs and MO‐EVs (Figure 7c). The differentially expressed microRNAs are identified in Supplementary Table 2.

### 3.6 TSA induced differential expression of EV microRNAs related to general regulation mechanisms and osteogenic differentiation

**FIGURE 8:**
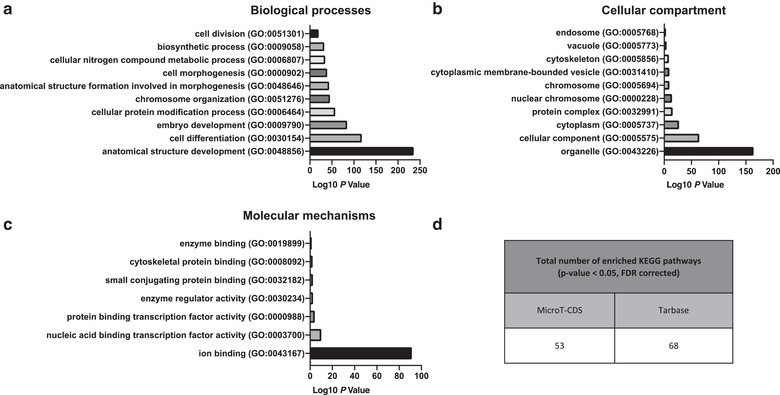
Gene ontology analysis of microRNAs found to be significantly upregulated in TSA‐EVs. Top ten GO prediction scores covering the domains of a) biological processes, b) cellular compartments and c) molecular mechanisms of microRNAs significantly upregulated in TSA‐EVs. d) Total number of experimentally validated and predicted interactions enrich KEGG pathways from MicroT‐CDS and Tarbase databases

To investigate the functions of the differentially expressed microRNAs, Gene ontology (GO) and Kyoto Encyclopaedia of Genes and Genomes (KEGG) pathways were assessed. MicroRNAs were found to be associated with GO functional annotation of biological processes (e.g., anatomical structural development, cell differentiation), cellular compartments (e.g., organelle, chromosome) and molecular mechanisms (e.g., ion binding, protein binding transcription factor) (Figure 8a‐c). The number of significantly affected KEGG pathways, analysed via experimentally validated and predicted interactions of the differentially expression EV microRNAs are shown in Figure 8d.

All significantly affected KEGG pathways, analysed by predicted and experimental validated interactions of the differentially expressed microRNAs related to osteogenic differentiation and general regulatory mechanisms, are summarised in Table 1. A total of 21 KEGG pathways directly or indirectly related to osteogenic differentiation were enriched within TSA‐EVs. Of note, enriched KEGG pathways of differentially expressed microRNAs including Hippo signalling (mmu04390), MAPK signalling (mmu04010), Wnt signalling (mmu04310), and ECM‐receptor interaction (mmu04512) pathways, are involved in osteogenic differentiation. Additionally, the differentially expressed microRNAs were enriched in pathways related to general regulatory mechanisms including regulation of actin cytoskeleton (mmu04810) and endocytosis (mmu04144), possibly signifying the role of these microRNAs in influencing the internalisation of EVs at the recipient cells. Moreover, enriched pathways of SNARE interactions in vesicular transport (mmu04130), mRNA surveillance pathway (mmu03015) and protein processing in endoplasmic reticulum (mmu04141) may indicate how these microRNAs modulate recipient cell function.

**TABLE 1:** Enriched KEGG biological pathways related to osteogenesis and regulatory mechanisms

KEGG pathways related to osteogenic differentiation		p‐value, FDR corrected	Genes	MiRNAs	Algorithms
Hippo signalling pathway	mmu04390	1.18E‐05	82	18	TarBase
		2.68E‐08	55	25	MicroT‐CDS
FoxO signalling pathway	mmu04068	3.75E‐07	85	19	TarBase
		1.04E‐03	54	28	MicroT‐CDS
PI3K‐Akt signalling pathway	mmu04151	1.32E‐03	111	29	MicroT‐CDS
Signalling pathways regulating pluripotency of stem cells	mmu04550	1.49E‐03	50	26	MicroT‐CDS
ECM‐receptor interaction	mmu04512	1.49E‐03	27	27	MicroT‐CDS
MAPK signalling pathway	mmu04010	1.71E‐04	129	18	TarBase
		1.96E‐03	86	30	MicroT‐CDS
cAMP signalling pathway	mmu04024	5.22E‐03	67	30	MicroT‐CDS
cGMP‐PKG signalling pathway	mmu04022	3.88E‐02	82	15	TarBase
		5.51E‐03	57	29	MicroT‐CDS
mTOR signalling pathway	mmu04150	1.95E‐03	38	19	TarBase
		7.73E‐03	25	22	MicroT‐CDS
Estrogen signalling pathway	mmu04915	2.09E‐02	49	15	TarBase
		1.09E‐02	29	25	MicroT‐CDS
AMPK signalling pathway	mmu04152	9.07E‐03	71	19	TarBase
		1.28E‐02	43	27	MicroT‐CDS
Focal adhesion	mmu04510	2.44E‐02	101	17	TarBase
		1.76E‐02	66	31	MicroT‐CDS
Insulin signalling pathway	mmu04910	1.60E‐03	78	18	TarBase
		1.82E‐02	48	27	MicroT‐CDS
TGF‐beta signalling pathway	mmu04350	1.60E‐03	43	18	TarBase
		2.00E‐02	32	24	MicroT‐CDS
Ras signalling pathway	mmu04014	4.56E‐02	103	19	TarBase
		2.03E‐02	63	30	MicroT‐CDS
Wnt signalling pathway	mmu04310	4.90E‐02	69	16	TarBase
		2.74E‐02	46	30	MicroT‐CDS
Hedgehog signalling pathway	mmu04340	3.32E‐02	20	23	MicroT‐CDS
Adherens junction	mmu04520	9.90E‐05	47	17	TarBase
TNF signalling pathway	mmu04668	6.48E‐04	58	16	TarBase
HIF‐1 signalling pathway	mmu04066	2.28E‐03	62	19	TarBase
Rap1 signalling pathway	mmu04015	2.16E‐02	102	18	TarBase

KEGG pathways related to general regulatory mechanism		p‐value, FDR corrected	Genes	MiRNAs	Algorithms
Sphingolipid signalling pathway	mmu04071	4.39E‐04	72	18	TarBase
		3.06E‐03	46	27	MicroT‐CDS
Regulation of actin cytoskeleton	mmu04810	9.07E‐03	108	18	TarBase
		3.22E‐03	72	31	MicroT‐CDS
Endocytotsis	mmu04144	6.12E‐07	128	18	TarBase
		7.67E‐03	67	29	MicroT‐CDS
SNARE interactions in vesicular transport	mmu04130	4.10E‐02	18	14	TarBase
mRNA surveillance pathway	mmu03015	4.37E‐02	31	23	MicroT‐CDS
Protein processing in endoplasmic reticulum	mmu04141	5.84E‐06	101	18	TarBase
		2.38E‐02	48	22	MicroT‐CDS

### 3.7 The proteome of TSA‐EVs is enriched in proteins involved in transcriptional regulation

**FIGURE 9:**
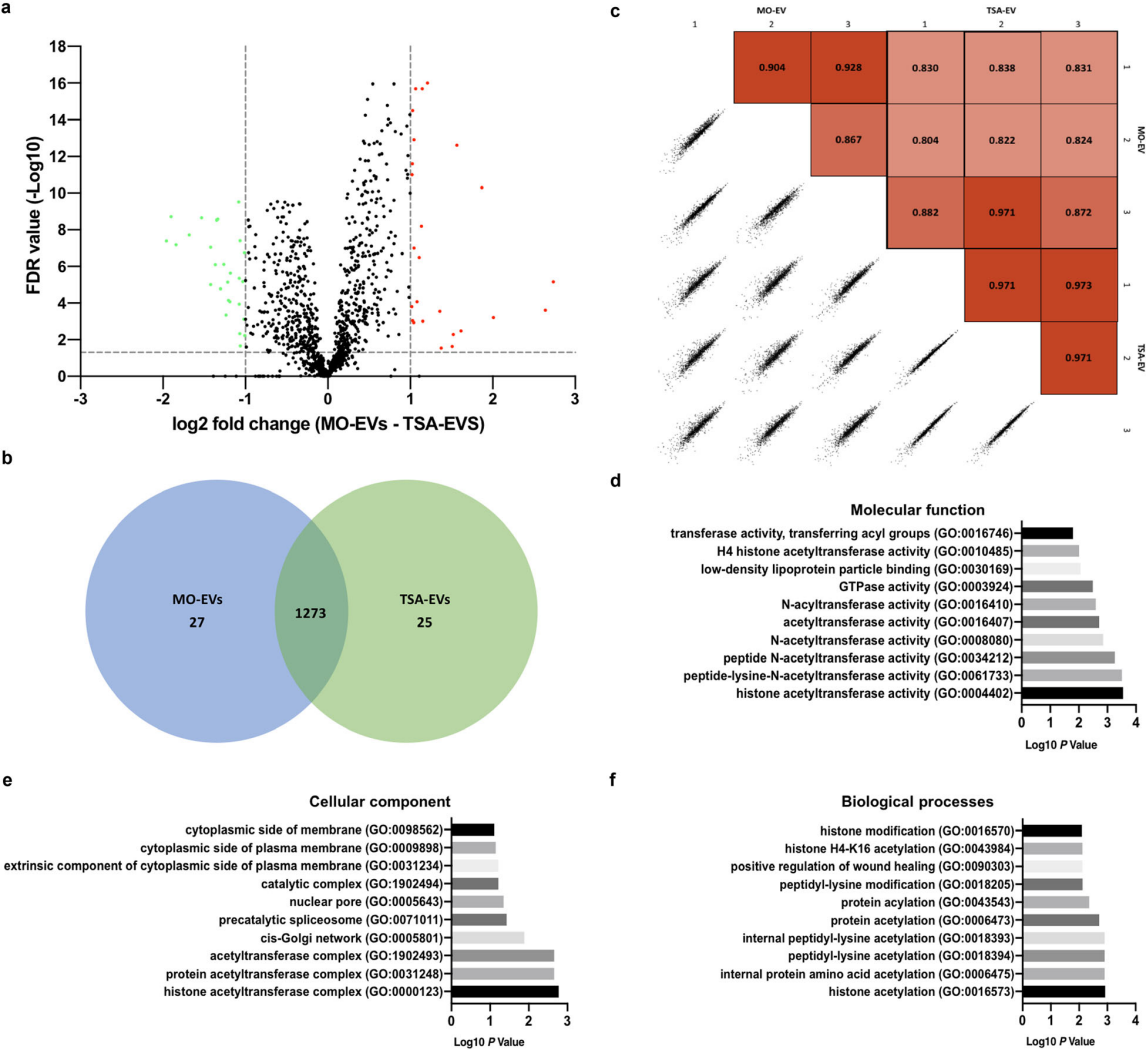
Analysis of differentially expressed proteins derived from TSA‐EVs and MO‐EVs. a) Volcano plot displaying Log_2_ values for the proteins fold‐change against Log_10_ FDR. Proteins with a Log_2_ fold difference below 1 and a statistical value of > 0.05 were not considered to be statistically significant (vertical and horizontal lines respectively). The red points in the plot represents the significantly upregulated TSA‐EV proteins, the green points represent significantly upregulated MO‐EVs proteins. b) Venn diagram comparing proteins differentially expressed from TSA‐EVs and MO‐EVs. A total of 1273 shared proteins; 25 proteins upregulated in TSA‐EVs and 27 proteins upregulated in MO‐EVs. c) Pearson correlations between technical replicates, biological replicates and sample groups were determined. GO analysis of proteins found to be significantly upregulated in TSA‐EVs. Top ten GO prediction scores covering the domains of d) molecular function, e) cellular components and f) biological processes of proteins significantly upregulated in TSA‐EVs

The proteomes of MO‐EVs and TSA‐EVs were compared for three independent sample preparations using label‐free MS‐LC/LC approach. The use of stringent criteria only permitted the inclusion of proteins identified in a least two biological replicates, with > 2 spectral counts in at least one repeat. Protein database searching resulted in the identification of a total of 1325 proteins. Of these, 25 proteins were significantly upregulated in TSA‐EVs, 27 upregulated in MO‐EVs, and 1273 shared proteins (Figure 9a, b). The differentially expressed proteins are identified in Supplementary Table 3. Pearson correlation, comparing all biological samples to one another, show a high average correlation between replicates in the MO‐EV (0.90) and TSA‐EV (0.97) groups (Figure 9c). When comparing MO‐EV and TSA‐EV to one another, an average correlation of 0.85 is observed, revealing a significant degree of similarity in the protein expression between MO‐EVs and TSA‐EVs. To provide an overview of the principal processes, mechanisms and cellular location of proteins significantly upregulated in TSA‐EVs, GO analysis was performed. Significantly enriched TSA‐EV proteins were found to be associated with GO functional annotation of molecular function (e.g., histone acetyltransferase activity, peptide‐lysine‐N‐acetyltransferase activity), cellular components (e.g., histone acetyltransferase complex, pre‐catalytic spliceosome) and biological processes (e.g., histone acetylation, histone modification) (Figure 9d‐f). The functional efficacy of TSA‐EVs on augmenting hBMSCs epigenetic functionality was evaluated by quantifying histone acetylation levels (Supplementary Figure 3). TSA‐EV treated hBMSCs elicited a significant increase in H3K9 acetylation levels in a time‐dependent manner when compared to the MO‐EVs treated (*P* ≤ 0.01 ‐ 0.001) and the untreated cells (*P* ≤ 0.001).

## 4 DISCUSSION

In the last decade, the field of EVs has rapidly expanded and is constantly unearthing new understanding of the role these nanoparticles play in various biological processes, signifying the potential influence EVs may have on the future direction of healthcare technologies. Several studies have demonstrated the therapeutic efficacy of EVs as novel acellular tools for bone repair (Davies et al., 2017; Wang et al., 2018; Wei et al., 2019). Although the potential utility of these nanosized vesicles has been reported, several approaches have been employed to further improve their therapeutic efficacy (Man et al., 2020). Genetic modification of the EV parent cell is a commonly utilised approach to promote the clinical viability of these nanoparticles (Kang et al., 2015; Tao et al., 2017); however, there are limitations associated with this technique (Hu et al., 2020; Kooijmans et al., 2016). Harnessing post‐translational modifications through altering the cells’ epigenetics has been demonstrated to enhance osteogenic differentiation (Hu et al., 2013; Huynh et al., 2016), hence, providing a potential novel strategy to improve EVs therapeutic efficacy for bone regeneration.

**FIGURE 10:**
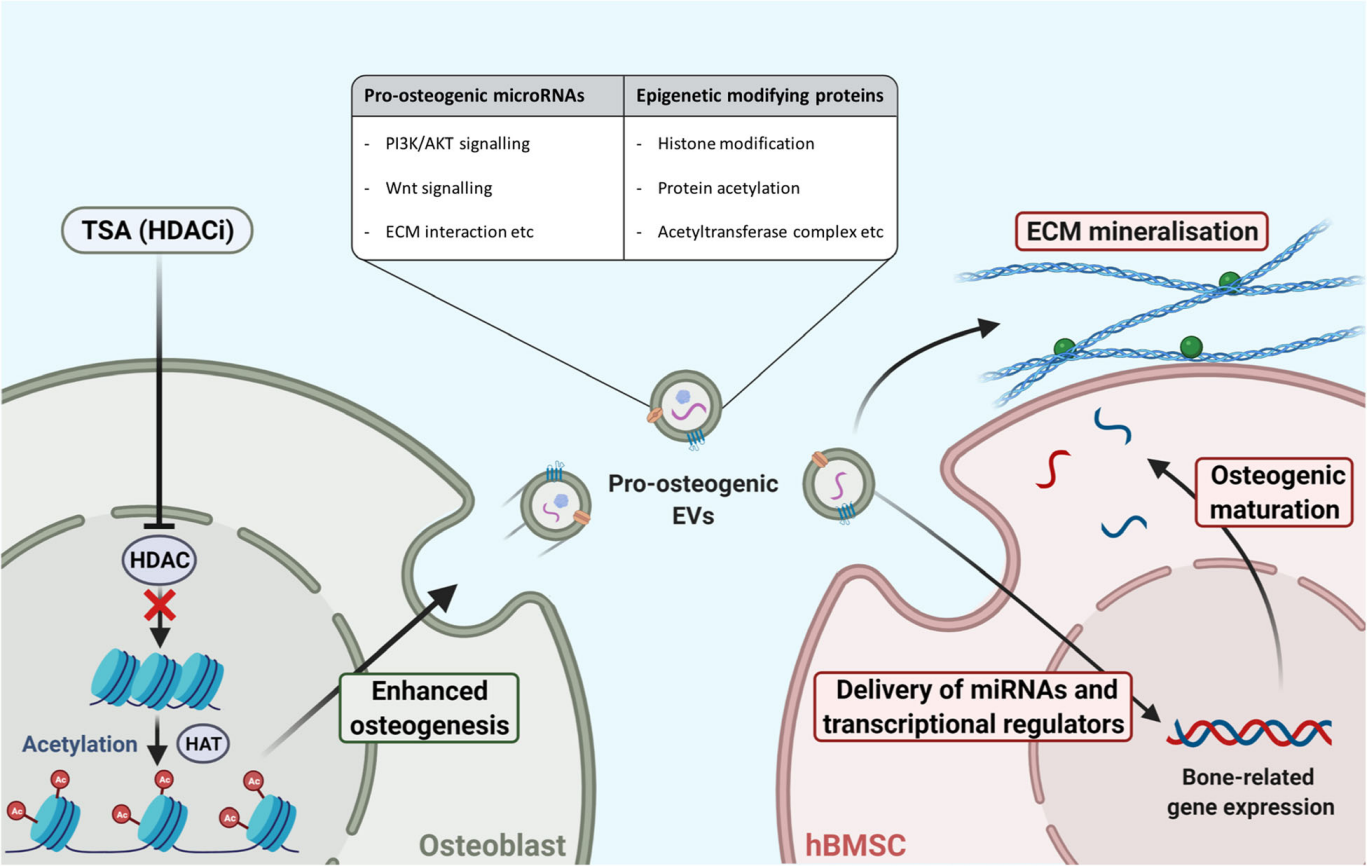
Schematic representation of the mechanism TSA augments osteoblast epigenetic functionality and mineralisation, enhancing the therapeutic potency of their secreted EVs to stimulate hBMSCs osteogenic differentiation. Figure created with BioRender.com

In the present study, we investigated the influence of altering osteoblast's epigenetic functionality to promote the osteoinductive capacity of their secreted EVs. TSA, a naturally‐derived HDACi, has been reported to accelerate osteogenic differentiation through hyperacetylation induced chromatin remodelling and transcription factor activation (Jin et al., 2013; Schroeder & Westendorf, 2005; Schroeder et al., 2004). Chromatin remodelling increases the accessibility to osteoblast‐related genes of interest, while HDAC inhibition and the hyperacetylation of non‐histone proteins promotes the activity/stability of key osteogenic transcriptional factors, such as Runx2 (Huynh et al., 2017). Herein, we demonstrated the successful augmentation of osteoblast epigenetic functionality through TSA‐induced histone hyperacetylation (Figure 10), consistent with several studies in the literature (Hu et al., 2013; Man et al., 2021; Schroeder & Westendorf, 2005). Additionally, we found that TSA induced acetylation resulted in a time‐dose dependent effect on osteoblast mineralisation, where administration of low TSA dosages (≤ 10 nM) increased osteogenesis when compared to higher concentrations (> 20 nM). This has been similarly reported in the literature (Huynh et al., 2016; Schroeder & Westendorf, 2005), likely due to the effects of high HDACi concentrations on the long‐term cellular viability. Hence, from a scalability perspective, administrating TSA at a dose that does not detrimentally affect cell number or functionality is highly advantageous (Rohde et al., 2019). In addition to examining the influence of TSA on osteoblast epigenetic functionality and differentiation, it is important to characterise the effects of this parental cell augmentation on the secreted EVs. It was observed that EVs isolated from TSA‐treated osteoblasts displayed reduced polydispersity (1.34‐fold), particle size (1‐05‐fold), concentration (1.4‐fold) and protein content (1.16‐fold) when compared to MO‐EVs. This profile has been reported to correlate with the degree of osteoblast differentiation (Davies et al., 2019), indicating the potentially enhanced osteoinductive capacity of TSA‐EVs. Moreover, the increased osteoblast extracellular matrix collagen production induced by TSA likely played a critical role in the quantity of EVs isolated from the conditional media, due to collagen‐mediated EV immobilisation (Buzás et al., 2018; Krohn et al., 2016). Interestingly, the TSA‐EVs exhibited a 3‐fold increase in RNA content when compared to that in the MO‐EVs. This suggests that the enhanced osteoblast transcriptional activity induced by TSA mediated hyperacetylation, enriched secreted EVs with elevated levels of RNA species, which may be partly responsible for its improved biological potency at recipient hBMSCs (Figure 10).

EVs ability to regulate intercellular communication such as stimulating the recruitment of endogenous cells is highly advantageous for bone tissue engineering applications (Su et al., 2018). Our findings demonstrate that osteoblast‐derived EVs significantly promoted hBMSCs proliferation and migration when compared to that of untreated cells, consistent with findings in the literature (Eichholz et al., 2020; Haertinger et al., 2020; Xu et al., 2019). Interestingly, TSA‐EVs elicited a more potent influence on hBMSCs proliferation and migration when compared to that of MO‐EVs. With TSA‐EVs exhibiting a 3‐fold increased RNA content compared to MO‐EVs, it is likely this enrichment plays a role in the enhanced cellular response to TSA‐EVs. It has been reported that microRNAs are the most abundant RNA species within EVs, likely due to their small size (Huang et al., 2013; Kim et al., 2017; O'brien et al., 2020). Hence, microRNA analysis was utilised to further elucidate the role of TSA in promoting osteoblast‐derived EVs paracrine effects. Intriguingly, the SNARE interactions in vesicular transport, endocytosis and regulation of actin cytoskeleton KEGG pathways were enriched by differentially expressed TSA‐EVs microRNAs, indicating the possible microRNA function in regulating EV internalisation at recipient cells. Moreover, TSA‐EVs were significantly enriched with miR‐31‐5p and miR‐143‐3p, microRNAs reported to promote the recruitment of numerous cells types (Li et al., 2015). Wang et al. demonstrated that miR‐143 targets HDAC7 in osteoblasts and endothelial cells, resulting in enhanced osteogenic and angiogenic effects, respectively (Wang et al., 2020). This indicates the role of miR‐143 in TSA‐EVs on promoting target cells differentiation capacity by modifying their epigenetic functionality. Therefore, the enrichment of these microRNAs in TSA‐EVs may be involved in promoting the recruitment and stimulation of endogenous cells through post‐translational modifications for bone regeneration.

Several studies have reported the use of HDACis in modifying the cells’ differentiation capacity (Huynh et al., 2016; Jin et al., 2013), however, there has been limited investigations into their influence in augmenting the cells secretome, particularly the therapeutic efficacy of EVs for bone regeneration. HDACis have been assessed as promising pharmacological agents to halt disease progression or for cancer therapeutics (Minetti et al., 2006; West & Johnstone, 2014), with their influence on EV secretion recently acquiring growing interest. For example, Sandonà et al. reported the utility of TSA in enhancing the therapeutic efficacy of EVs derived from fibro‐adipogenic progenitors (FAPs) to support muscle stem cells‐mediated regeneration of dystrophic muscles. HDACi treatment enriched FAP‐EVs with miR‐206 leading to enhanced regeneration and reduced fibrosis (Sandonà et al., 2020). Wang et al. used of the HDACi B390 to induce apoptosis in pancreatic cancer cells and decreased vascular endothelial growth factor C expression in secreted EVs, inhibiting the nanoparticles role in cancer progression (Wang et al., 2020). Herein, we investigated the influence of epigenetic reprogramming through TSA induced hyperacetylation, on the osteoinductive potency of osteoblast‐derived EVs as a novel engineering approach. We demonstrated that EVs isolated from TSA‐treated osteoblasts substantially promoted the mineralisation capacity of hBMSCs through the early, mid and late‐stages of osteogenic differentiation exhibited by enhanced osteoblast‐related gene/protein expression, ALP activity and extracellular matrix mineralisation compared to MO‐EVs treated and the untreated cells. Additionally, TSA‐EVs elicited an enhanced dose‐dependent increase in hBMSCs extracellular matrix collagen and calcium deposition when compared to MO‐EV treatment, thus providing further evidence into TSA‐EVs increased osteoinductive properties. Interestingly, the MO‐EVs treated hBMSCs presented a disparity in the differentiation status of the cell when compared to the osteogenic maturity of the mineralised matrix. Our findings showed a slight non‐significant increase in the MO‐EV treated hBMSCs osteoblast‐related gene/intracellular protein expression, while also exhibiting a significantly enhanced ALP activity, extracellular matrix collagen production and calcium deposition when compared to the untreated control. This indicates the reduced efficacy of MO‐EVs in stimulating hBMSCs differentiation compared to TSA‐EVs, however, MO‐EVs were able to facilitate the mineralisation of the cell's extracellular matrix, likely due to the enrichment of pro‐mineralising proteins associated with MO‐EVs, including Annexins as reported in the literature and in the proteomics analysis in this present study (Davies et al., 2017, 2019). Moreover, the more mature collagen extracellular matrix from the TSA‐EV treated hBMSCs, likely promoted the sequestering of endogenous EVs secreted from differentiating hBMSCs and TSA‐EV treatment (Buzás et al., 2018; Krohn et al., 2016), thus further facilitating matrix mineralisation. Taken together, the functional analysis of TSA‐EVs osteoinductive efficacy indicates its ability to promote osteogenesis through stimulating hBMSCs osteogenic differentiation and the mineralisation of its extracellular matrix (Figure 10).

To further elucidate the mechanisms in which TSA‐EVs imparts its pro‐osteogenic effects, particularly in stimulating cellular differentiation, microRNA profiling was conducted. Utilising available databases, target prediction of differentially expressed TSA‐EV microRNAs showed substantial enrichment in KEGG pathways related to osteogenic differentiation such as Hippo signalling pathway, MAPK signalling pathway, Wnt signalling pathway, FoxO signalling pathway and ECM‐receptor interaction. MicroRNA profiling highlighted several pro‐osteogenic microRNAs significantly enriched in the TSA‐EVs. Among them, miR‐21 is known to target Spry1, an inhibitor of osteogenic differentiation, and Sox2, a marker of pluripotency, hence resulting in the osteogenic lineage‐specific differentiation (Li et al., 2015; Yang et al., 2013). Furthermore, miR‐21 is involved in the regulation of the phosphatidylinositol 3‐kinase (PI3K)/Akt/glycogen synthase kinase‐3β (GSK‐3β) signalling pathway, leading to an increased accumulation of cytoplasmic β‐catenin, enhanced Runx2 expression and osteogenic differentiation (Meng et al., 2015). Similarly, reports have shown that miR‐26a promotes osteogenic differentiation by targeting GSK‐3β, activating the Wnt signalling pathway (Liu et al., 2018; Su et al., 2015). Zhao et al. demonstrated that miR‐199b‐5p expression was increased during osteogenesis, and when overexpressed it promoted BMSCs osteogenic differentiation through GSK3β/β‐catenin pathway (Zhao et al., 2016). Additionally, miR‐15b, an osteoblast‐specific microRNA (Sainitya et al., 2015), is known to target Smurf1, an inhibitor of osteogenic differentiation (Vimalraj et al., 2014). Smurf1 degrades Runx2 through the proteasomal pathway, therefore, the upregulation of miR‐15b will indirectly increase Runx2 levels (Shimazu et al., 2016). Another miRNA upregulated in TSA‐EVs, miR‐181a, has been reported to promote osteogenesis via inhibiting key transforming growth factor‐β (TGF‐β) signalling molecules such as the TGF‐β type I receptor (TβR‐I/Alk5) and TGF‐β induced (Bhushan et al., 2013; Zheng et al., 2019). Moreover, miR‐125b, a microRNA found to be involved in osteoblast‐osteoclast communication was significantly enriched in TSA‐EVs (Yoshiko & Minamizaki, 2020). Minamizaki et al. reported miR‐125b involvement in regulating bone turnover, where the enrichment of this microRNA in EVs within the bone matrix, inhibits bone resorption via downregulating the transcriptional repressor PRDM1, subsequently upregulating the anti‐osteoclastogenic genes *Mafb* and *Irf8* (Minamizaki et al., 2020). Interestingly, TSA‐EVs were significantly enriched in miR‐22, which has been reported to inhibit the expression of HDAC6 in adipose‐derived stem cells, promoting its osteogenic differentiation (Huang et al., 2012). Similarly, miR‐143 has been shown to stimulate BMSCs and osteoblast mineralisation by inhibiting HDAC7 activity (Wang et al., 2020). This indicates that modifying the osteoblast epigenome through TSA, augments the epigenetic functionality of recipient hBMSCs through EV microRNA delivery, mimicking the mechanism of transgenerational epigenetic inheritance reported in the literature (Sahoo & Losordo, 2014; Sharma, 2014). Together, these findings suggest altering the epigenetic functionality and differentiation of mineralising osteoblast enriches their EVs with pro‐osteogenic microRNAs (Figure 10). Future studies would be required to functionally validate the biological efficacy of these differentially expressed microRNAs. Moreover, it would be of interest to examine the influence on TSA induced epigenetic regulation on the expression and therapeutic efficacy of other RNAs species within the TSA‐EVs.

In order to further investigate the influence of TSA induced epigenetic modification on the composition and biological potency of TSA‐EVs, the vesicles protein composition was analysed via mass spectrometry. GO analysis showed that the differentially expressed TSA‐EV proteins were associated with transcriptional regulation. Among them, Ankyrin repeat domain‐containing protein 11 (AnkRD11) is a chromatin regulator which modulates histone acetylation and gene expression (Gallagher et al., 2015). The introduction of a N‐ethyl‐N‐nitrosourea mutation in the AnkRD11 gene in mice, resulted in craniofacial abnormalities and reduced overall bone mineral density, indicating the role of this protein in normal skeletal development (Barbaric et al., 2008). Another chromatin regulator, KAT8 regulatory NSL complex subunit 3 (Kansl3) was found to be upregulated in TSA‐EVs. Kansl3 is a subunit of the histone acetyltransferase complex, which is involved in histone H4 acetylation and thereby regulating transcriptional activity (Cai et al., 2010). Moreover, histone H4 acetylation has been reported to promote osteogenic maturation, indicating the lineage‐specific effects of Kansl3 on hBMSCs differentiation (Dudakovic et al., 2013). Similarly, the Plant homeodomain finger protein 14 (PHF14) was significantly enriched in TSA‐EVs. Several studies have reported the role of PHD fingers as epigenetic effectors by regulating transcription and chromatin dynamics (Aasland, 1995; Musselman & Kutateladze, 2011) and their role on osteogenesis has been reported in the literature. For example, the PHF20 was shown to positively regulate osteoblast differentiation by increasing H3K4me3 enrichment on the Runx2 promoter, enhancing the transcription factor activity (Yang et al., 2017). In addition to chromatin remodelling proteins, TSA‐EVs were also significantly enriched with proteins involved in regulating RNA processing. For example, the pre‐mRNA‐splicing factor 38B (Prpf38b) was found to be upregulated in TSA‐EVs. Pre‐mRNA splicing factors has been shown to mediate mRNA processing and splicing (Di et al., 2019), indicating the role of Prpf38b in regulating the translation of pre‐mRNA transcripts to mature mRNA in hBMSCs. Similarly, RNA‐binding protein 10 (Rbm10) is known to be involved in mRNA processing and miRNA biogenesis (Zhao et al., 2017). The nuclear carrier protein Ran‐binding protein 17 (Ranbp17) was also enriched in TSA‐EVs, which functions in regulating the transport of proteins and RNAs through the nuclear pore (Lee et al., 2010). Similarly, Washc5 has been shown to mediate endosomal sorting, indicating its role in processing endocytosed EVs (Gilleron et al., 2019). Interestingly, the pro‐osteogenic proteins previously identified in MO‐EVs to be responsible for its pro‐mineralising capacity (Davies et al., 2017, 2019), such as calcium‐channelling annexin proteins, were not significantly altered by TSA treatment. The TSA induced enrichment of EV proteins involved in transcriptional regulation indicates the role of TSA‐EVs in augmenting the epigenetic landscape of the recipient cell (Figure 10), confirmed by the functional assessment of TSA‐EV induced hBMSCs histone hyperacetylation. The delivery of these transcriptional regulating proteins likely facilitate the therapeutic efficacy of TSA‐EV pro‐osteogenic microRNAs and ultimately accelerates the osteogenic maturation of hBMSCs. Moreover, the augmented epigenetic landscape induced by TSA‐EV transcriptional modifiers probably facilitated the enhanced hBMSCs expression of osteoblast‐related genes during the early phase of osteogenesis. It is important to note that due to the diverse biological cargo of EVs, is it likely that the osteoinductive capacity of TSA‐EVs is a combination of changes across all EV components (i.e. metabolites, lipids, proteins, RNA species etc.), although this would require further investigation.

In the present study, we investigated the influence of altering the epigenetic functionality of mineralising osteoblasts on enhancing the therapeutic efficacy of their EVs for bone regeneration. We isolated and characterised EVs from TSA‐treated mineralising osteoblasts and further examined the biological potency of these EVs on hBMSCs. Having demonstrated the enhanced osteoinductive capacity of TSA‐EVs, microRNA profiling and proteomic analysis was conducted to further elucidate the possible mechanism in which these nanoparticles promote osteogenesis. We predicted the targets of the TSA‐EV enriched microRNAs and the signalling pathways to be related to osteogenic differentiation. Moreover, the TSA‐EVs were significantly enriched with transcription regulating proteins. Hence, the TSA‐EV enrichment of these pro‐osteogenic microRNAs and proteins involved in epigenetic regulation may be partially responsible for promoting hBMSCs osteogenic differentiation, however, the functional validation of these augmented components induced by epigenetic reprogramming would be required in future studies.

Having reported the enhanced osteoinductive capacity of TSA‐EVs in vitro, there is growing precedence to elucidate the therapeutic capacity of EVs in more physiologically relevant models in vivo (Man et al., 2020). There are several pertinent issues with the therapeutic delivery of EVs in the field including controlling their release kinetics at the defect site, optimising EV dosing regimen to maximise their therapeutic response in vivo and the development of a biomaterial system that facilitates EV‐induced bone regeneration (Brennan et al., 2020). We believe these factors would have a tremendous impact on the clinical efficacy of EVs, irrespective of the nanoparticle's therapeutic potency. Although the in vivo investigation of TSA‐EVs is beyond the scope of this present study, which is focused on determining whether epigenetic reprogramming is a viable EV engineering approach and elucidating possible mechanisms linked to its enhanced osteoinductive properties, future studies will assess the therapeutic delivery of TSA‐EVs within a biomaterial system to effectively investigate the clinical efficacy of this EV engineering approach for bone augmentation strategies. In addition to conducting in vivo testing, to move this novel approach towards the clinic, demonstration of TSA‐EVs efficacy against a positive treatment control would be a valuable pursuit, for example BMP2.

## 5 CONCLUSION

In conclusion, these findings demonstrate that altering osteoblasts epigenetic functionality via TSA induced hyperacetylation, enhanced the differentiation capacity of the parental cell and the osteoinductive potency of their EVs. Furthermore, microRNA profiling revealed HDACi treatment enriched TSA‐EVs with microRNAs associated with osteogenic‐related pathways. Proteomics analysis identified the enrichment of epigenetic regulating proteins within TSA‐EVs. These findings demonstrate the considerable utility of epigenetic regulation as a novel engineering approach to enhance the therapeutic efficacy of EVs as an acellular tool for bone repair. To our knowledge, this is the first study to promote EVs regenerative potency for bone augmentation strategies through epigenetic reprogramming.

## CONFLICTS OF INTEREST

The authors declare no conflicts of interest.

## AUTHOR CONTRIBUTIONS

Kenny Man study conceptualisation, biological laboratory work and manuscript preparation. Mathieu Y. Brunet dynamic light scattering analysis. Soraya Williams sample preparation for proteomic analysis and immunoblotting. Maria Fernandez‐Rhodes immunoblotting. Liam M. Heaney design and planning of proteomic analysis. Lee A. Gethings mass spectrometry analysis. Angelica Federici TEM imaging. Owen G. Davies, David Hoey and Sophie C. Cox contributed with critical revisions and editing. All authors have read and agreed to the published version of the manuscript.

## Supporting information

Supporting information.
