# Supplementary material for: Epigenetic reprogramming enhances the therapeutic efficacy of osteoblast‐derived extracellular vesicles to promote human bone marrow stem cell osteogenic differentiation
Source: J Extracell Vesicles. 2021 Jul 7;10(9):e12118. doi: 10.1002/jev2.12118 (PMC8263905; doi:10.1002/jev2.12118)
Supplement: Supplementary file 1 — Supporting information. [file JEV2-10-e12118-s001.docx]

**Supplementary Figures**

**
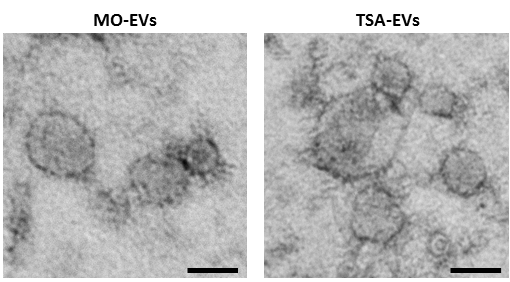
**

**Supplementary Figure 1. Transmission electron microscopy of osteoblast-derived EVs.** Scale bar = 50 nm.


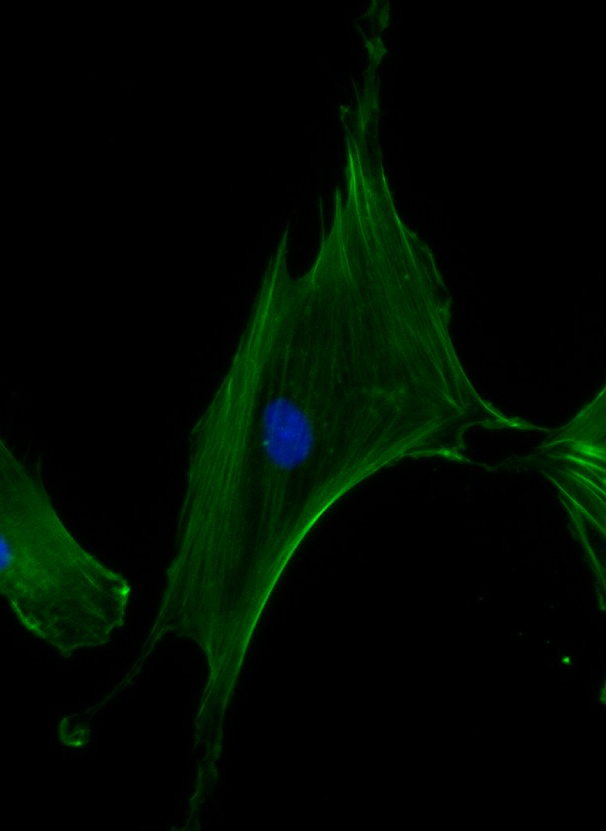


**Control**


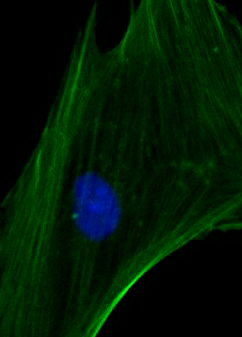


**Actin**

**DAPI**

**Cell Mask**


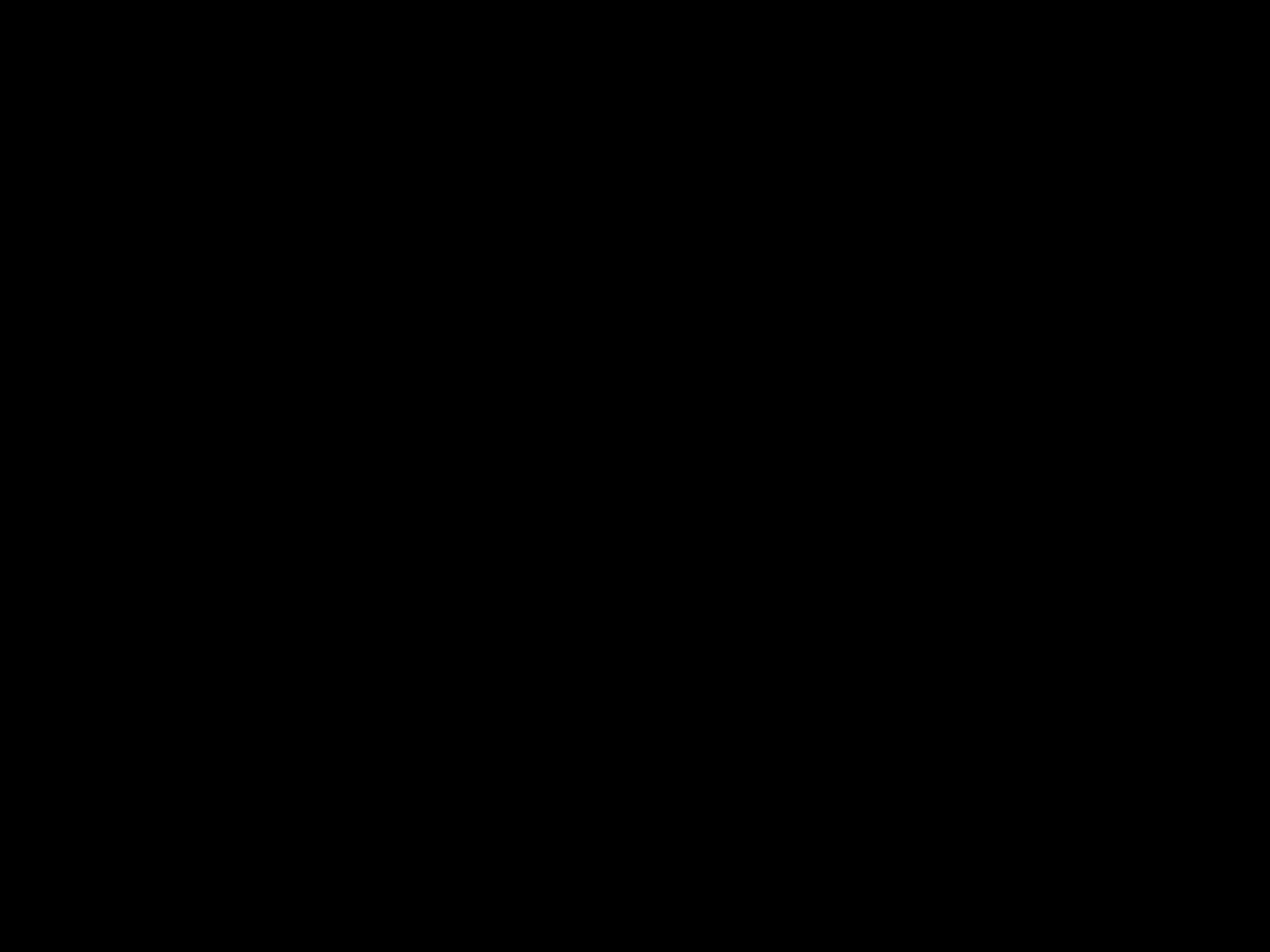


**Cell Mask**

**Supplementary Figure 2. Immunofluorescent images of non-EV treated hBMSCs.** Scale bar = 20 µm.

**Supplementary Figure 3. The effects of TSA-EV treatment on hBMSCs histone acetylation levels.** TSA-EVs altered hBMSCs H3K9 histone acetylation levels in a time-dependant manner. Data are expressed as mean ± SD (n=3). **P ≤ 0.01 and ***P ≤ 0.001.

**Supplementary Table 1.** Primer sequences used for RT-qPCR

| **Gene** | **Forward** | **Reverse** |
| --- | --- | --- |
| *ALP* | CTTGGGCAGGCAGAGAGTA | AGTGGGAGGGTCAGGAGAT |
| *COL1A1* | AGACAGTGATTGAATACAAAACCA | GGAGTTTACAGGAAGCAGACA |
| *BSP* | GAGGTGATAGTGTGGTTTATGGA | TGATGTCCTCGTCTGTAGCA |
| *OCN* | GGCACCCTTCTTTCCTCTTC | TTCTGGAGTTTATTTGGGAGCA |

**Supplementary Table 2.** Differentially expressed TSA-EVs microRNAs

| **miRNA_ID_List** | **ID** | **Fold changes** | **p values** |
| --- | --- | --- | --- |
| mmu-miR-133b-5p | 188 | 2.22 | 2.11E-03 |
| mmu-miR-30b-5p | 99 | 3.71 | 2.01E-06 |
| mmu-miR-199a-3p | 291 | 2.12 | 1.52E-04 |
| mmu-miR-125b-5p | 163 | 2.09 | 2.22E-04 |
| mmu-miR-181a-5p | 246 | 2.84 | 1.31E-03 |
| mmu-miR-31-5p | 109 | 2.47 | 1.70E-02 |
| mmu-miR-3963 | 1122 | 2.45 | 1.92E-02 |
| mmu-miR-451a | 542 | 4.22 | 1.96E-02 |
| mmu-miR-16-5p | 45 | 2.83 | 2.00E-02 |
| mmu-miR-30c-5p | 102 | 3.49 | 2.10E-02 |
| mmu-miR-199b-5p | 293 | 2.35 | 2.31E-02 |
| mmu-miR-199a-5p | 292 | 3.02 | 2.37E-02 |
| mmu-miR-21a-5p | 62 | 2.78 | 2.88E-02 |
| mmu-miR-5100 | 1134 | 3.79 | 3.08E-02 |
| mmu-miR-195a-5p | 284 | 3.95 | 3.09E-02 |
| mmu-miR-26a-5p | 78 | 2.74 | 3.51E-02 |
| mmu-miR-143-3p | 213 | 3.60 | 3.52E-02 |
| mmu-miR-140-3p | 206 | 2.66 | 3.78E-02 |
| mmu-miR-181b-5p | 249 | 2.57 | 4.04E-02 |
| mmu-miR-152-3p | 236 | 3.69 | 1.15E-03 |
| mmu-miR-30e-5p | 106 | 2.81 | 1.14E-03 |
| mmu-miR-1983 | 967 | 2.16 | 1.12E-02 |
| mmu-miR-15b-5p | 42 | 3.34 | 1.66E-02 |
| mmu-miR-130a-3p | 178 | 4.53 | 1.20E-03 |
| mmu-miR-22-3p | 65 | 2.68 | 3.12E-02 |
| mmu-miR-148a-3p | 226 | 4.02 | 4.46E-02 |
| mmu-miR-26b-5p | 80 | 2.23 | 4.65E-02 |
| mmu-miR-7012-5p | 1547 | -2.02 | 8.59E-05 |
| mmu-miR-1966-5p | 953 | -2.42 | 2.82E-02 |
| mmu-miR-6385 | 1257 | -2.27 | 2.31E-03 |
| mmu-miR-3099-3p | 1061 | -2.07 | 1.58E-02 |
| mmu-miR-8119 | 1911 | -2.30 | 6.88E-03 |
| mmu-miR-6978-5p | 1479 | -3.87 | 2.03E-03 |

**Supplementary Table 3.** Differentially expressed TSA-EVs proteins

| **Gene Name** | **Protein** | **Accession** | **Log_2_ fold change** | **p-value** |
| --- | --- | --- | --- | --- |
| **Ranbp17** | Ran-binding protein 17 | Q99NF8 | 1.207 | 1.11E-16 |
| **F2** | Prothrombin | P19221 | 1.145 | 2.22E-16 |
| **Rps3a** | 40S ribosomal protein S3a | P97351 | 1.066 | 2.22E-16 |
| **Mrps25** | 28S ribosomal protein S25_ mitochondrial | Q9D125 | 1.027 | 3.33E-15 |
| **Kdelr2** | ER lumen protein-retaining receptor 2 | Q9CQM2 | 1.046 | 1.39E-13 |
| **Kansl3** | KAT8 regulatory NSL complex subunit 3 | A2RSY1 | 1.565 | 2.78E-13 |
| **Crp** | C-reactive protein | P14847 | 1.025 | 2.72E-12 |
| **Niban1** | Protein Niban 1 | Q3UW53 | 1.022 | 1.07E-11 |
| **Ganc** | Neutral alpha-glucosidase C | Q8BVW0 | 1.868 | 5.57E-11 |
| **Myadm** | Myeloid-associated differentiation marker | O35682 | 1.136 | 6.41E-09 |
| **Tomm70** | Mitochondrial import receptor subunit TOM70 | Q9CZW5 | 1.045 | 9.89E-08 |
| **Sgpl1** | Sphingosine-1-phosphate lyase 1 | Q8R0X7 | 1.109 | 3.22E-07 |
| **Rbm10** | RNA-binding protein 10 | Q99KG3 | 2.735 | 7.08E-06 |
| **Dnajb6** | DnaJ homolog subfamily B member 6 | O54946 | 1.083 | 8.48E-05 |
| **Krt74** | Keratin_ type II cytoskeletal 74 | Q6IFZ9 | 1.018 | 1.58E-04 |
| **Ern1** | Serine/threonine-protein kinase/endoribonuclease IRE1 | Q9EQY0 | 2.639 | 2.53E-04 |
| **Ankrd11** | Ankyrin repeat domain-containing protein 11 | E9Q4F7 | 1.358 | 2.80E-04 |
| **Prpf38b** | Pre-mRNA-splicing factor 38B | Q80SY5 | 2.010 | 6.28E-04 |
| **Gnaz** | Guanine nucleotide-binding protein G(z) subunit alpha | O70443 | 1.029 | 9.20E-04 |
| **Washc5** | WASH complex subunit 5 | Q8C2E7 | 1.153 | 1.01E-03 |
| **Rap2c** | Ras-related protein Rap-2c | Q8BU31 | 1.043 | 1.17E-03 |
| **Krt28** | Keratin_ type I cytoskeletal 28 | A6BLY7 | 1.616 | 3.35E-03 |
| **Gmip** | GEM-interacting protein | Q6PGG2 | 1.522 | 5.29E-03 |
| **Ctps2** | CTP synthase 2 | P70303 | 1.508 | 2.40E-02 |
| **Phf14** | PHD finger protein 14 | Q9D4H9 | 1.375 | 2.96E-02 |
| **Rab31** | Ras-related protein Rab-31 | Q921E2 | -1.082 | 3.04E-10 |
| **Retsat** | All-trans-retinol 13_14-reductase | Q64FW2 | -1.903 | 1.99E-09 |
| **Krt7** | Keratin_ type II cytoskeletal 7 | Q9DCV7 | -1.530 | 2.24E-09 |
| **Sphkap** | A-kinase anchor protein SPHKAP | Q6NSW3 | -1.335 | 2.72E-09 |
| **Rpl7a** | 60S ribosomal protein L7a | P12970 | -1.347 | 2.98E-09 |
| **Pds5a** | Sister chromatid cohesion protein PDS5 homolog A | Q6A026 | -1.682 | 1.89E-08 |
| **Fam3c** | Protein FAM3C | Q91VU0 | -1.065 | 4.00E-08 |
| **Prdx6** | Peroxiredoxin-6 | O08709 | -1.959 | 4.16E-08 |
| **Rpl9** | 60S ribosomal protein L9 | P51410 | -1.840 | 6.71E-08 |
| **Espl1** | Separin | P60330 | -1.420 | 9.15E-08 |
| **Sri** | Sorcin | Q6P069 | -1.011 | 1.81E-07 |
| **Fermt2** | Fermitin family homolog 2 | Q8CIB5 | -1.262 | 7.71E-07 |
| **Sun2** | SUN domain-containing protein 2 | Q8BJS4 | -1.366 | 8.16E-07 |
| **Src** | Neuronal proto-oncogene tyrosine-protein kinase Src | P05480 | -1.182 | 2.32E-06 |
| **Cacnb1** | Voltage-dependent L-type calcium channel subunit beta-1 | Q8R3Z5 | -1.074 | 4.62E-06 |
| **Tdrd7** | Tudor domain-containing protein 7 | Q8K1H1 | -1.032 | 6.75E-06 |
| **Flot1** | Flotillin-1 | O08917 | -1.214 | 7.29E-06 |
| **Dcaf6** | DDB1- and CUL4-associated factor 6 | Q9DC22 | -1.421 | 1.00E-05 |
| **Atp1b3** | Sodium/potassium-transporting ATPase subunit beta-3 | P97370 | -1.303 | 1.71E-05 |
| **Map3k12** | Mitogen-activated protein kinase kinase kinase 12 | Q60700 | -1.205 | 7.17E-05 |
| **Unc13d** | Protein unc-13 homolog D | B2RUP2 | -1.187 | 8.57E-05 |
| **Topaz1** | Protein TOPAZ1 | E5FYH1 | -1.074 | 1.18E-04 |
| **Tm9sf4** | Transmembrane 9 superfamily member 4 | Q8BH24 | -1.235 | 4.69E-04 |
| **Ripor1** | Rho family-interacting cell polarization regulator 1 | Q68FE6 | -1.017 | 7.73E-04 |
| **Psmb1** | Proteasome subunit beta type-1 | O09061 | -1.068 | 4.71E-03 |
| **Plg** | Plasminogen | P20918 | -1.007 | 6.04E-03 |
| **Gipc2** | PDZ domain-containing protein GIPC2 | Q9Z2H7 | -1.060 | 2.23E-02 |
